# β‐Adrenergic Signaling Promotes Anti‐Tumor Immunity in TP53‐mutant Oral Squamous Cell Carcinoma

**DOI:** 10.1002/advs.202516859

**Published:** 2026-02-10

**Authors:** Frederico O. Gleber‐Netto, Deborah Silverman, Tongxin Xie, Shamima Akhter, Nicole R. Vaughn, Adewale Adebayo, Kala Chand Debnath, Shorook Naara, Shajedul Islam, Erik Knutsen, Emily Lorin Ashkin, Simone Anfossi, Sara Leahey, Patrick Hwu, Sebastien Talbot, Erica K Sloan, Robert Saddawi‐Konefka, Roberto Rangel, Jeffrey N. Myers, George A. Calin, Moran Amit

**Affiliations:** ^1^ Department of Head and Neck Surgery The University of Texas MD Anderson Cancer Center Houston TX USA; ^2^ Department of Leukemia and Division of Cancer Medicine The University of TX MD Anderson Cancer Center Houston TX USA; ^3^ Department of Medical Biology UiT The Arctic University of Norway Tromsø Norway; ^4^ Department of Cancer Immunology and Immunotherapy Moffitt Cancer Center Tampa FL USA; ^5^ Department of Translational Molecular Pathology The University of Texas MD Anderson Cancer Center Houston TX USA; ^6^ Department of Pharmacology and Physiology Karolinska Institute Stockholm Sweden; ^7^ Monash Institute of Pharmaceutical Sciences Monash University Parkville Victoria Australia; ^8^ Department of Cancer Biology The University of Texas MD Anderson Cancer Center Houston TX USA

**Keywords:** β‐adrenergic signaling, CXCL10, head and neck cancer, neuroscience

## Abstract

Head and neck squamous cell carcinoma (HNSCC) is notoriously resistant to immunotherapy. The interplay between β‐adrenergic signaling and p53 loss, both key regulators of immune responses, has remained largely unexplored in the setting of tumor‐immune evasion. This study demonstrates that pharmacologic stimulation of β2‐adrenergic receptors with isoprenaline significantly enhances cytotoxic T cell activity against p53‐deficient HNSCC cells via a CXCL10‐dependent paracrine mechanism. Comprehensive transcriptomic and co‐culture assays reveal that p53‐null cancer cells upregulate CXCL10, which promotes CD8^+^ T cell recruitment and activation. Neutralization of CXCL10 abolishes the β‐adrenergic‐induced cytotoxic T cell response, establishing this chemokine as a pivotal mediator. Using tyrosine hydroxylase knockout mouse models, we show that adrenergic innervation is essential for intra‐tumoral CXCL10 expression and the infiltration of effector CXCR3^+^ T cells in vivo. Notably, the CXCL10‐driven T cell response is associated with simultaneous upregulation of both activation and exhaustion markers, indicating a robust but transient effector state within the tumor microenvironment. Collectively, these findings uncover a neuro‐immune axis that reverses immune escape in p53‐deficient HNSCC and suggest novel therapeutic strategies targeting adrenergic signaling to convert immune “cold” tumors into “hot” ones more amenable to immunotherapy.

## Graphical abstract

β‐adrenergic stimulation enhances anti‐tumor immunity in TP53‐deficient oral squamous cell carcinoma by inducing tumor‐derived secretion of CXCL10, which attracts and activates cytotoxic CD8^+^ T cells. The findings demonstrate that β‐adrenergic signaling alters tumor–immune interactions via CXCL10‐mediated paracrine activation, revealing a neuro‐immune axis that overcomes immune escape in p53‐deficient tumors.

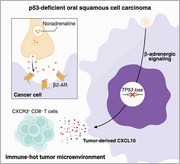

## 1 Introduction

Head and neck squamous cell carcinoma (HNSCC) remains largely refractory to immunotherapeutic interventions, with most patients exhibiting primary resistance and a lack of predictive biomarkers for therapeutic response [1]. A defining feature of HNSCC is the high prevalence of TP53 mutations, which occur in approximately 75% of cases and profoundly shape the clinical heterogeneity and immune landscape of this malignancy [2]. The absence of functional p53 not only rewires secretory profiles but also profoundly alters the tumor microenvironment, resulting in marked differences in the release of inflammatory mediators and the regulation of immune cell recruitment when compared to p53–competent tumors [1, 2, 3, 4, 5, 6, 7]. Notably, mutant TP53 disrupts key cytokine pathways, such as TNFα‐mediated signaling, further accentuating immune evasion [8, 9, 10, 11]. Our prior findings demonstrated that the loss of *TP53* leads to enhanced adrenergic innervation within tumors [12].

Recent research has highlighted the autonomic nervous system, particularly β‐adrenergic signaling, as a potent regulator of both cancer progression and tumor immunity [13, 14, 15, 16, 17, 18, 19, 20]. β2‐adrenergic receptors (β2‐ARs) are expressed on both malignant and immune cells, and several prior studies have shown that direct β‐adrenergic stimulation of immune cells typically exerts suppressive effects on their anti‐tumor activity. Clinically, β‐blocker use in HNSCC patients undergoing immunotherapy has been linked with diminished therapeutic responses and worse survival, reinforcing the paradigm of adrenergic‐mediated immunosuppression in this context [20]. Yet, β‐adrenergic signaling also influences tumor‐intrinsic pathways, such as those controlled by p53, and its role within the complex tumor microenvironment is incompletely understood [21].

While TP53 mutant tumors are generally considered immunosuppressed, a subset paradoxically displays immune‐enriched phenotypes, suggesting underlying heterogeneity in neuro‐immune regulation. Here, we specifically investigate the indirect regulation of anti‐tumor immunity: we test the impact of β‐adrenergic stimulation not on immune cells directly, but on TP53‐deficient HNSCC cells and their secretome, and how this, in turn, affects immune cell function and recruitment. Our study deciphers the interplay between adrenergic signals, p53 status, and the immune compartment, with a focus on the modulation of the tumor immune microenvironment through paracrine, tumor cell‐mediated mechanisms.

## 2 Results

### 2.1 β‐Adrenergic Receptors Are Prevalent in HNSCC and Are Associated with the Characteristics of the Tumor Microenvironment

**FIGURE 1:**
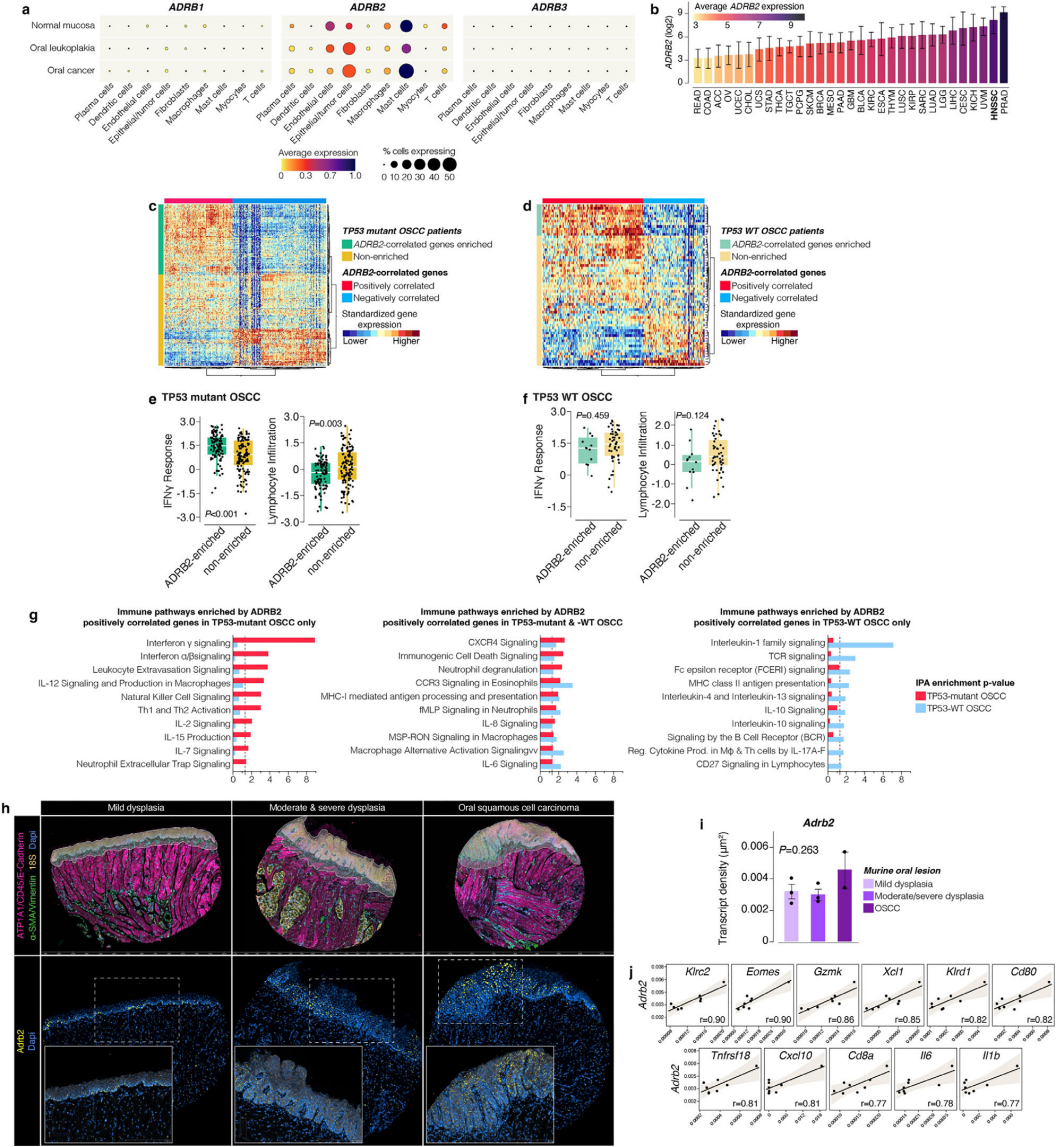
Beta adrenergic receptors expression and its correlation with immune genes. a) Average expression and percentage of cells expressing *ADRB1*, *ADRB2*, and *ADRB3* genes in cells from oral mucosa from normal, oral premalignant lesions (leukoplakia), and oral squamous cell carcinoma samples from the GSE181919 dataset [22]. b) *ADRB2* RNA expression in solid tumors from The Cancer Genome Atlas [24]. Bar plots and error bars represents *ADRB2* mean expression and standard deviation (mean ± SD), for the following tumor cohorts: rectum adenocarcinoma (READ, n = 160): 3.21 ± 1.18; colon adenocarcinoma (COAD, n = 449): 3.24 ± 1.33; adrenocortical carcinoma (ACC, n = 79): 3.51 ± 1.43; ovarian serous cystadenocarcinoma (OV, n = 305): 3.61 ± 1.2; uterine corpus endometrial carcinoma (UCEC, n = 532): 3.66 ± 1.71; cholangiocarcinoma (CHOL, n = 36): 3.73 ± 1.58; uterine carcinosarcoma (UCS, n = 57): 4.36 ± 1.5; stomach adenocarcinoma (STAD, n = 415): 4.52 ± 1.56; thyroid carcinoma (THCA, n = 505): 4.64 ± 1.12; testicular germ cell tumors (TGCT, n = 134): 4.71 ± 0.77; pheochromocytoma and paraganglioma (PCPG, n = 179): 4.76 ± 1.32; skin cutaneous melanoma (SKCM, n = 103): 5.07 ± 1.62; breast invasive carcinoma (BRCA, n = 1097): 5.17 ± 1.35; mesothelioma (MESO, n = 87): 5.19 ± 1.16; pancreatic adenocarcinoma (PAAD, n = 178): 5.23 ± 1.29; lymphoid neoplasm diffuse large B‐cell lymphoma (DLBC, n = 48): 5.43 ± 1.48; glioblastoma multiforme (GBM, n = 155): 5.47 ± 1.15; bladder urothelial carcinoma (BLCA, n = 408): 5.53 ± 1.82; kidney renal clear cell carcinoma (KIRC, n = 533): 5.64 ± 0.93; esophageal carcinoma (ESCA, n = 184): 5.71 ± 2.12; thymoma (THYM, n = 120): 5.86 ± 1.29; lung squamous cell carcinoma (LUSC, n = 502): 6.07 ± 1.54; kidney renal papillary cell carcinoma (KIRP, n = 290): 6.08 ± 1.57; sarcoma (SARC, n = 259): 6.22 ± 1.73; lung adenocarcinoma (LUAD, n = 515): 6.25 ± 1.43; brain lower grade glioma (LGG, n = 515): 6.26 ± 1.08; liver hepatocellular carcinoma (LIHC, n = 371): 6.78 ± 1.75; cervical squamous cell carcinoma and endocervical adenocarcinoma (CESC, n = 305): 7.06 ± 2.14; kidney chromophobe (KICH, n = 66): 7.19 ± 1.72; uveal melanoma (UVM, n = 80): 7.29 ± 1.12; head and neck squamous cell carcinoma (HNSC, n = 520): 8.12 ± 1.72; and prostate adenocarcinoma (PRAD, n = 497): 9.12 ± 0.74. Data are presented as log2‐transformed average gene expression per tumor group. Error bars represent group standard deviation. c) The heatmaps show hierarchical clustering analysis considering *ADRB2*‐correlated genes (Pearson's correlation coefficient ≤‐0.4 or ≥ 0.4) among *TP53* mutant OSCC cases. d) The heatmaps show hierarchical clustering analysis considering *ADRB2*‐correlated genes (Pearson's correlation coefficient ≤‐0.4 or ≥ 0.4) among *TP53* wild type (WT) OSCC cases. e) Boxplots showing the enrichment of immune‐related signatures among the groups of patients shown in panel c. Differences were evaluated employing a two‐tailed Student's t‐test under the assumption of equal variances. f) Boxplots showing the enrichment of immune‐related signatures among the groups of patients shown in panel d. Differences were evaluated employing a two‐tailed Student's t‐test under the assumption of equal variances. g) The bar plots illustrate selected immune‐related canonical pathways that are enriched exclusively by *ADRB2*‐positively correlated genes in *TP53* mutant OSCC, exclusively in *TP53* wild type (WT) OSCC, and in both categories. h) Upper row shows hematoxylin & eosin (H&E) stained slides of oral mucosa tissue obtained from mice treated with 4‐NQO for oral carcinogenesis induction, showing premalignant (oral dysplasia) and malignant (oral squamous cell carcinoma—OSCC) lesions. The bottom row shows the expression of Adrb2 in the same tissues measured by spatial transcriptome analysis. i) Quantification of *Adrb2* expression in the epithelial region of the tissue shown in h, according to the tissue histology. Differences were assessed using one‐way ANOVA. j) The scatterplots illustrate the correlation between *Adrb2* expression and immune‐related genes within the same samples as in h. The Pearson Correlation test was employed to calculate the correlation coefficient among genes.

To assess the presence of β‐adrenergic receptors in oral tissues, we conducted an in silico analysis using published single‐cell RNA sequencing (scRNA‐seq) data from normal oral epithelium, oral premalignant lesions (oral leukoplakia), and oral squamous cell carcinoma[8]. Our results showed that *ADRB2* is widely expressed in cells of the oral mucosa, regardless of pathological condition. In contrast, *ADRB1* was detected at very low levels, with fewer than 3% of each cell type expressing the gene. *ADRB3* was not present in any oral mucosa cells (Figure 1a). Considering the overall *ADRB2* expression across each tissue type, tumor cells accounted for the majority of *ADRB2* expression in OSCC, representing 51.4% of all *ADRB2*‐expressing cells. In oral leukoplakia and normal oral mucosa, epithelial cells were responsible for 15% and 5.5% of *ADRB2*‐expressing cells, respectively, indicating that *ADRB2* expression may be upregulated in oral keratinocytes during the carcinogenesis process. These findings imply that *ADRB2* is the predominant receptor mediating β‐adrenergic signaling in oral mucosa cells, thereby directing our focus toward this receptor to investigate the role of β‐adrenergic signaling in oral carcinogenesis.

To assess the expression level of *ADRB2* in HNSCC relative to other human malignancies, we analyzed The Cancer Genome Atlas (TCGA) RNA sequencing data[9]. The results showed that HNSCC samples have the second‐highest average *ADRB2* expression among all TCGA tumors, indicating a potential role in oral carcinogenesis (Figure 1b).

We previously established that loss of p53 activity impacts adrenergic tumor innervation in HNSCC, being associated with increased adrenergic innervation within the tumor microenvironment [12]. Besides influencing β‐adrenergic signaling, *TP53* mutations are also associated with alterations in the immune response of HNSCC. Gain‐of‐function *TP53* mutations were associated with a distinct cytokine profile that hinders cytotoxic T cell infiltration, thereby weakening the anti‐tumor immune response [6]. In this way, we investigated whether the relationship between *ADRB2* expression and the presence of immune‐related molecular features differs between *TP53*‐mutant and wild‐type (WT) oral squamous cell carcinoma (OSCC) samples from The Cancer Genome Atlas (TCGA) [23, 25]. *ADRB2* expression was significantly correlated (correlation coefficient ≤‐0.4 or ≥0.4, p < 0.05) with 374 genes in *TP53*‐mutant and 230 genes in *TP53*‐WT OSCC cases (Table S1). Among these, 49 genes were significantly correlated with *ADRB2* in both *TP53*‐mutant and WT groups. Hierarchical clustering analysis (HCA) of these *ADRB2*‐correlated genes revealed a subset of patients exhibiting enrichment of *ADRB2* and its correlated genes in both *TP53*‐mutant and WT OSCC groups (Figure 1c,d). To determine whether the expression of *ADRB2*‐correlated genes was associated with distinct tumor immune features between *TP53*‐mutant and WT OSCC, we compared the enrichment of immune‐related signatures (*as previously outlined* [25]) between these groups. We found reduced Lymphocyte Infiltration and an increase in IFN‐γ Response signatures in *ADRB2*‐correlated gene signature‐enriched *TP53*‐mutant OSCC, whereas these changes were not evident among *TP53*‐WT cases (Figure 1e,f).

To understand the differences between the *ADRB2*‐correlated gene signatures from *TP53*‐mutant and wild‐type (WT) groups, we conducted pathway analysis (using Ingenuity Pathway Analysis – IPA) considering only genes positively correlated (correlation coefficient CC ≥ 0.2 and p‐value < 0.05) in one group but not in the other (CC ≤ 0.15 and p‐value ≥ 0.05), subsequently. *ADRB2*‐correlated genes only in the *TP53*‐mutant OSCC group were predominantly associated with cytokine signaling (n = 23, 40.3%), innate immunity (n = 19, 33.3%), and inflammation (n = 5, 8.7%) pathways (Figure 1g and Table S2). Conversely, genes correlated with *ADRB2* only in the *TP53*‐WT OSCC group showed a higher association with adaptive immunity pathways (n = 5, 13.9%) and were less associated with inflammatory processes (n = 1, 2.8%). Only 11 pathways were enriched in both gene sets (6.3% of pathways enriched in *TP53*‐mutant and 4% in the *TP53*‐WT group), indicating that these two gene sets are associated with distinct immune‐related signals (Figure 1g and Table S2). These findings corroborate our hypothesis that the expression of *ADRB2* is correlated with specific tumor immune molecular characteristics, contingent upon the mutation status of *TP53* in OSCC. This suggests that the functional status of tumor p53 may modulate the impact of β‐adrenergic signaling on oral cancer immunity.

To further explore the link between *ADRB2* expression and immunity in the context of *TP53* mutant tissues, we analyzed oral mucosa samples from p53‐null mice subjected to oral carcinogenesis induced by 4‐NQO treatment. We performed spatial transcriptome analysis (Xenium In situ) on premalignant and malignant oral lesions from this mouse model, examining the distribution of β‐adrenergic receptors in these tissues. To focus our analysis on the epithelial component, we delineated the regions of interest focusing on the oral epithelium and subjacent lamina propria and measured *Adrb2* transcript density (Figure S1). We observed abundant *Adrb2* expression in the oral keratinocytes of both premalignant and malignant lesions (Figure 1h; Figure S1). Although *Adrb2* expression varied among samples and along the epithelial lining, its density did not differ with the degree of epithelial transformation (Figure 1i).

To verify whether *Adrb2* expression was correlated with the expression of immune‐related transcripts, we analyzed the correlation between *Adrb2* density and the expression of the 478 genes in our custom Xenium gene panel. *Adrb2* density was positively correlated with 48 genes, mostly related to immune response (n = 28) (Figure 1j) and neuronal signaling (n = 12) (Table S3). These included genes linked to the function and modulation of cytotoxic T cells and Natural Killer cells (*Klrc1, Klrc2, Kldr1, Ncr1, Cd8a, Gzmk, Eomes*), immune co‐regulatory molecules (*Pdcd1lg2, Pdcd1, Cd80, Tnfrsf18)*, and cytokines (*Cxcl10, Il6, Il1b, Ccl6, Cxcl9, Ccl5, Xcl1*). Notably, several of these genes were also positively correlated with *ADRB2* in human *TP53*‐mutant OSCC samples from TCGA, including *PDCD1LG2* (PD‐L2), *CD274* (PD‐L1), and *CXCL10*. These findings show that *Adrb2* is abundantly expressed in malignant and premalignant oral keratinocytes in this mouse model, and they confirm that *Adrb2* expression is linked to immune‐related gene expression in the oral mucosa during oral carcinogenesis.

### 2.2 Pharmacological Stimulation of Adrenergic Signaling Increases Anti‐Tumor T Cell Cytotoxicity Against TP53‐Null Tumors but Not Against TP53‐WT Tumors

Based on these findings, we conducted an in vitro assay to investigate the impact of β‐adrenergic signaling on the anti‐tumor immune response in OSCC with varying *TP53* statuses. For this, we used a T‐cell‐mediated killing assay[11] with *TP53*‐isogenic PCI13 OSCC cell lines as a model. The PCI13 cell line lacks p53 protein activity due to a homozygous deletion in the *TP53* gene. In our study, we used PCI13 cell lines that were transduced with either a WT *TP53* (*TP53* ^+/+^) vector to restore its p53 activity or a control vector (*TP53* ^−/−^) [27, 28].

T‐cell‐mediated cytotoxicity was measured by co‐culturing CD8^+^ T cells with *TP53* ^+/+^ PCI13 or *TP53^−/−^* PCI13 cells, using cleaved caspase‐3 staining to detect apoptosis. To facilitate antigen recognition, *TP53* ^+/+^ and *TP53* ^−/−^ PCI13 cells were stably transduced with *HLA‐A2* and pulsed with the MART‐1 (Melanoma Antigen Recognized by T‐cells 1) peptide, thereby enhancing the recognition of cancer cells by T cells and improving the rate of T‐cell‐mediated cytotoxicity against these cells. Then, they were co‐cultured with HLA‐matched, MART‐1‐specific CD8+ T cells.

**FIGURE 2:**
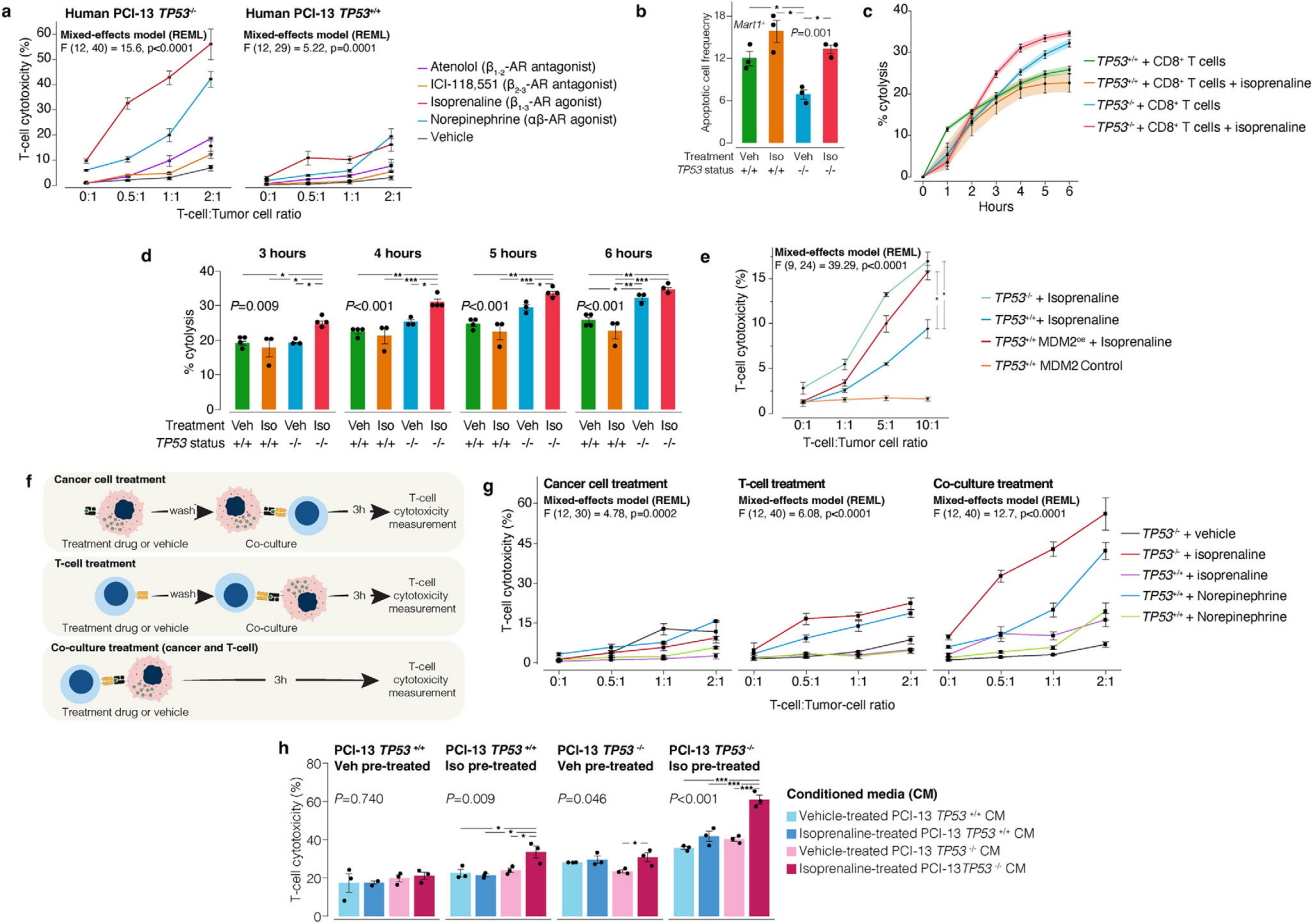
In vitro assessment of the implications of beta‐adrenergic signaling modulation in head and neck cancer cells concerning their susceptibility to T cell‐mediated killing. a) *TP53* ^+/+^ and *TP53^−/−^* PCI13 cells co‐cultured with HLA‐matched, MART‐1‐specific human CD8+ T cells, in the presence of adrenergic agonists or blockers, for 3 h, before flow cytometry assessment of caspase‐3 activity. Data show T cell‐mediated cytotoxicity, represented as a percentage of PCI‐13 cells expressing cleaved caspase‐3. Error bars represent the standard error of the mean. Differences were assessed using a mixed‐effects model with restricted maximum likelihood (REML) estimation; post hoc comparisons at specific T‐cell: tumor cell ratios were evaluated using Tukey's test. b) Live cell analyzer (IncuCyte) cytotoxicity assay for *TP53* ^+/+^ and *TP53^−/−^* PCI13 cells (HLA‐A2+MART‐1+) co‐cultured with human MART‐1‐specific CD8+ T cells, in the presence or absence of isoprenaline for 3 h. Data are represented as a percentage of apoptotic cells (cleaved caspase‐3/7 positive). ANOVA followed by the Tukey‐Kramer HSD test for pairwise comparison; *P < 0.05, **P < 0.001, ***P < 0.0001. c) Long‐term cell death monitoring assay based on continuous assessment of cytotoxicity based on impedance measurement. *TP53* ^+/+^ and *TP53^−/−^* PCI13 cells co‐cultured with activated Hu PB CD8+ T cells. Data represents the percentage of cytolysis (the difference in the impedance between the parental cell line—*TP53* ^+/+^ or *TP53^−/−^* PCI13 cells monoculture—and each experimental group—cancer cell with/without T cell co‐culture and isoprenaline treatment—divided by the parental cell line impedance values) along 6 h following the start of the experiment. d) The bar plots represent the comparison of the cytolysis rate among each experimental group for 3, 4, 5, and 6 h. ANOVA followed by the Tukey‐Kramer HSD test for pairwise comparison; *P < 0.05, **P < 0.001, ***P < 0.0001. e) *TP53* ^−/−^, *TP53* ^+/+^, or *TP53* ^+/+^MDM2‐overexpressing PCI‐13 cells (HLA‐A2+MART‐1+) were co‐cultured with human MART‐1‐specific CD8+ T cells, in the presence or absence of isoprenaline. Data show T cell‐mediated cytotoxicity, represented as a percentage of PCI‐13 cells expressing cleaved caspase‐3. Differences were assessed using a mixed‐effects model with restricted maximum likelihood (REML) estimation; post hoc comparisons at specific T‐cell: tumor cell ratios were evaluated using Tukey's test. *P < 0.05. Error bars represent the standard error of the mean. f) Different methodologies for drug administration conditions in the evaluation of T cell‐mediated cytotoxicity against cancer cells. g) T cell‐mediated cytotoxicity in *TP53^−/−^* PCI13 cells exposed to 3 distinct approaches of drug administration (*as shown in* f), measured by flow cytometry assessment of caspase‐3 activity. Data is represented as a percentage of cells expressing cleaved caspase‐3. Differences were assessed using a mixed‐effects model with restricted maximum likelihood (REML) estimation; post hoc comparisons at specific T‐cell: tumor cell ratios were evaluated using Tukey's test. Error bars represent the standard error of the mean. *P < 0.05. h) T cell‐mediated cytotoxicity in *TP53^+/+^* and *TP53^−/−^* PCI13 cells pretreated with vehicle control or isoprenaline exposed to conditioned media (CM) from *TP53^+/+^* or *TP53^−/−^* PCI13 cells exposed to isoprenaline or vehicle, measured by flow cytometry assessment of caspase‐3 activity. Data is represented as a percentage of cells expressing cleaved caspase‐3. One‐way ANOVA followed by the Tukey‐Kramer HSD test for pairwise comparison; *P < 0.05, **P < 0.001, ***P < 0.0001.

For in vitro modulation of β‐adrenergic signaling in OSCC cancer cells, the agonist isoprenaline (ISO), the nonspecific agonist norepinephrine (NEP), and the antagonists ICI 551 and atenolol (ATE) were used. Treatment with ISO or NEP markedly elevated apoptosis in *TP53* ^−/−^ PCI13 cells compared to the vehicle. In contrast, the changes observed in *TP53* ^+/+^ cells (Figure 2a) were significantly less pronounced, indicating that β‐adrenergic stimulation enhances T‐cell cytotoxicity specifically in p53‐deficient cells.

To validate these findings, we employed a distinct caspase‐3 and caspase‐7 killing assay (Sartorius IncuCyte system) using the same *TP53*‐isogenic cell lines and focusing only on the effect of isoprenaline. Treatment with ISO did not notably alter the apoptotic rate in *TP53* ^+/+^ PCI13 cells; however, it did enhance the apoptotic rate in *TP53* ^−/−^ PCI13 cells (Figure 2b). These changes did not occur when the cells were exposed to the same conditions without T‐cells, ruling out the possibility that ISO directly caused cancer cell death (Figure S2). These findings indicate that while β‐adrenergic stimulation does not impair the ability of T‐cells to target p53‐competent cancer cells, it does augment their effectiveness against p53‐deficient tumor cells.

A third validation experiment utilized the same cancer cells in a Maestro Edge device to facilitate long‐term monitoring of cell death through continuous impedance measurements. ISO‐treated cancer cells were co‐cultured for several hours with activated Hu PB CD8+ T cells. Results showed that cytolysis in *TP53* ^−/−^ PCI13 cells increased after 3 h compared to other groups, but this effect began to decline by the sixth hour, likely due to isoprenaline oxidation (Figure 2c). As previously noted, ISO‐treated *TP53* ^−/−^ PCI13 cells showed a significantly higher rate of cytolysis than vehicle‐treated *TP53* ^−/−^ cells and ISO‐ or vehicle‐treated *TP53* ^+/+^ cells at 3‐, 4‐, and 5‐h post‐co‐culture (Figure 2d). These results further indicate that β‐adrenergic signaling boosts T cell‐mediated toxicity against p53‐deficient HNSCC cells, but not against those with functional p53.

Next, we sought to clearly define the role of p53 activity in the observed effect on ISO‐induced enhancement of T‐cell‐mediated cancer cell killing. To achieve this, we overexpressed MDM2 in *TP53* ^+/+^ PCI13 cells (Figure S3). MDM2 is an E3 ubiquitin ligase that negatively regulates p53 by promoting its degradation through ubiquitination [29]. Overexpression of MDM2 made *TP53* ^+/+^ PCI13 cells more susceptible to T‐cell‐mediated cytotoxicity upon ISO treatment, similar to *TP53* ^−/−^ PCI13 cells (Figure 2e), confirming the significance of p53 loss in the enhanced T‐cell‐mediated killing induced by ISO.

Our results so far suggest that β‐adrenergic signaling in cancer cells plays a vital role in enhancing T‐cell cytotoxicity. However, the direct effect of ISO on β‐AR in T cells may also contribute to the observed phenotype. Existing literature indicates that β‐adrenergic signaling can inhibit T‐cell cytotoxicity [15, 30, 31, 32, 33]. Therefore, the increase in T‐cell cytotoxicity observed in our experiments is unlikely to be caused by isoprenaline's direct action on T cells. To explore this, we performed an additional T‐cell‐mediated killing assay in which ISO was administered solely to either T cells or cancer cells. A washing step was performed after this treatment, before mixing the cells in a co‐culture system, to ensure that no isoprenaline was carried over. We then compared these groups with the standard ISO administration method in co‐cultured cancer and T cells (Figure 2f). Our results showed that isoprenaline‐mediated T‐cell cytotoxicity was more pronounced in the co‐culture setting (Figure 2g). This indicates that the ISO‐mediated increase in T‐cell cytotoxicity against p53‐deficient HNSCC cells may be due to secondary molecules released into the medium.

Based on these findings, we hypothesize that ISO activates β2‐adrenergic receptors on OSCC cells, which in turn promotes paracrine signaling with T cells, thereby enhancing T cell‐mediated killing. To evaluate this, conditioned media (CM) were produced from *TP53* ^−/−^ and *TP53* ^+/+^ PCI13 cell monocultures treated with either isoprenaline (10 µM for 48 h) or a vehicle control. Concurrently, *TP53* ^−/−^ and *TP53* ^+/+^ PCI13 cancer cells were subjected to a 24‐h treatment with isoprenaline or vehicle, followed by washing and co‐culturing with HLA‐matched, antigen‐specific CD8+ T cells. These co‐cultures were subsequently treated with one of the four prepared CMs and incubated for 3 h before undergoing apoptosis assessment using cleaved caspase‐3 staining. In co‐cultures where cancer cells were pre‐treated with a vehicle control, the tumor's apoptotic rate was consistent across PCI‐13 *TP53* ^+/+^ cells that received various CMs. There was only a slight difference in the tumor's apoptotic rate between *TP53* ^−/−^ cells that received CM from vehicle‐treated *TP53* ^−/−^ cells and those that received CM from isoprenaline‐treated *TP53* ^−/−^ cells (Figure 2h). Conversely, in co‐cultures where cancer cells received pre‐treatment with isoprenaline, CM derived from isoprenaline‐treated *TP53* ^−/−^ cells induced a significant increase in the rate of tumor cell apoptosis in both *TP53* ^−/−^ and *TP53* ^+/+^ PCI13 cells (Figure 2h). These findings suggest that the CM from ISO‐treated p53‐null cells, but not from *TP53* ^+/+^ cells, can boost T cell‐mediated killing of cancer cells, regardless of the *TP53* status of the cancer cell treated with the CM. This supports our hypothesis that the increased T cell cytotoxicity caused by ISO is driven by paracrine signaling initiated by p53‐null OSCC cells. However, as this effect was observed solely when cancer cells were pre‐treated with isoprenaline, in addition to augmenting paracrine signaling, β‐adrenergic stimulation may also induce supplementary alterations in the cancer cells that favor an increase in T‐cell‐mediated cytotoxicity. The transfer of isoprenaline with the CM could constitute a potential confounding factor in this assay. However, it has been empirically demonstrated that isoprenaline undergoes oxidation in cell culture media within a matter of hours [34]. Most importantly, any residual isoprenaline should be comparable in the conditioned media derived from both *TP53* ^−/−^ and *TP53* ^+/+^ cells. Nevertheless, the cytotoxic effect remained significantly more pronounced in co‐cultures with conditioned media from p53‐deficient cells.

### 2.3 β‐Adrenergic Stimulation Promotes Cytokine Production by Oral Cancer Cells

**FIGURE 3:**
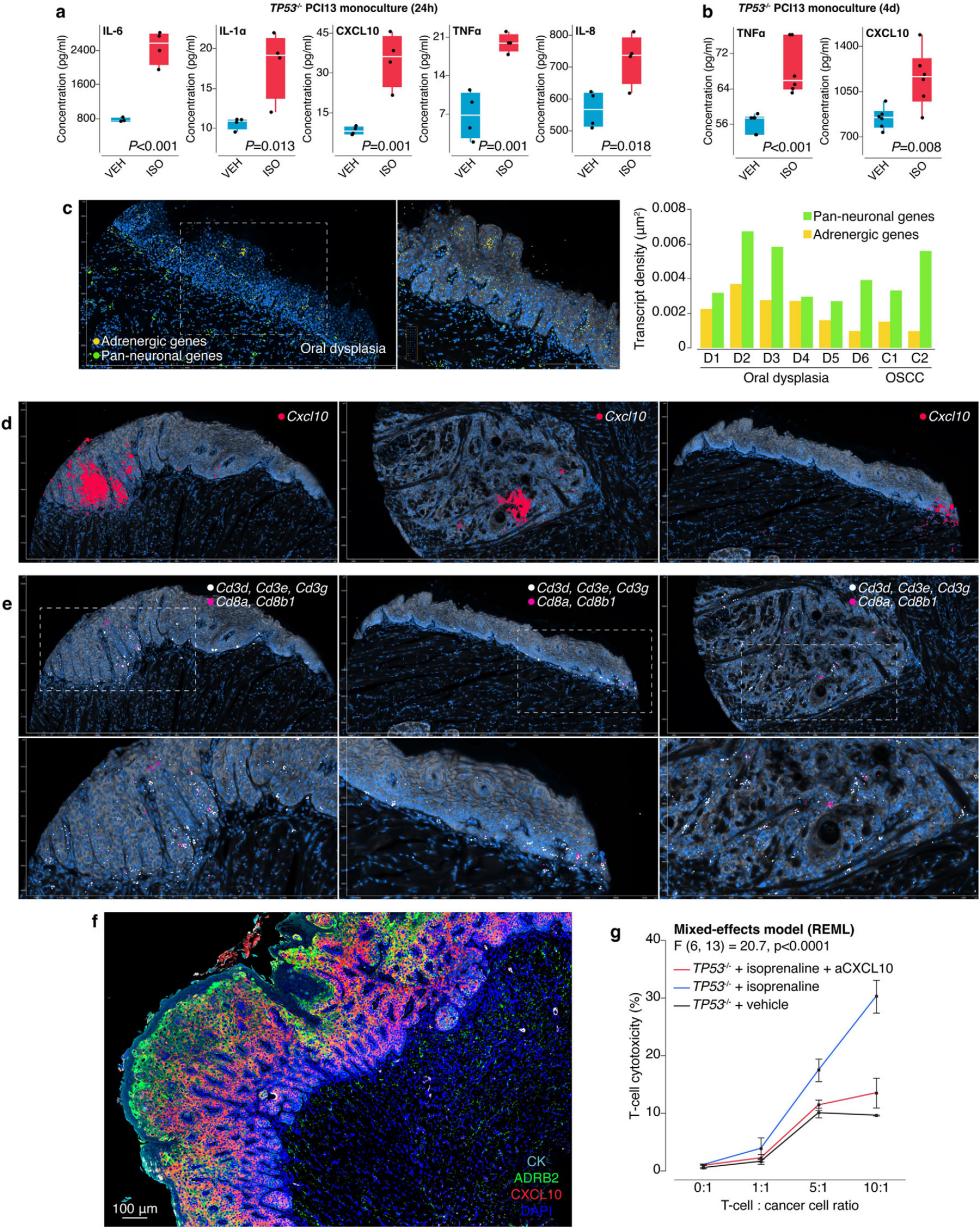
Assessment of how β‐adrenergic stimulation influences cytokine production in p53‐deficient HNSCC cancer cells. a) Assessment of cytokine concentration in the CM of *TP53^−/−^* PCI13 cells treated or not with isoprenaline for 24 h. Differences were evaluated employing a two‐tailed Student's t‐test under the assumption of equal variances. b) Assessment of cytokine concentration in the CM of *TP53^−/−^* PCI13 cells treated or not with isoprenaline for 4 days. Differences were evaluated employing a two‐tailed Student's t‐test under the assumption of equal variances. c) Assessment of peripheral nerve fibers and their adrenergic subgroup in the oral mucosa lesions from 4‐NQO‐treated animals by spatial transcriptome analysis. Peripheral (*Calca, Cdh19, Gap43, Gfra1, Gpr37l1, Lgi4, Mag, Plp1, Pmp22, Uchl1*) and adrenergic (*Dbh, Scl18a2, Slc6a2*) nerve fibers were detected based on the expression of nerve‐specific genes. The bar plot displays transcript density for all genes within each signature, per µm^2^, considering only the epithelial and adjacent lamina propria layers from each sample analyzed. d) Expression of *Cxcl10* in the oral lesions from 4‐NQO‐treated animals by spatial transcriptome analysis. e) Expression of T cell markers (Cd3d, Cd3e, Cd3g) and CD8+ T cell markers (Cd8a, Cd8b1) in the same samples with elevated Cxcl10 levels, as shown in the panel *d*. Observe the increased density of these markers surrounding regions exhibiting elevated Cxcl10 expression. f) Multiplex immunofluorescence was employed to detect pan‐cytokeratin (CK) (cyan), ADRB2 (green), and CXCL10 (red) proteins in oral mucosa lesions from animals treated with 4‐NQO. Note the co‐expression of CXCL10 and ADRB2 in the oral keratinocytes along the oral mucosa. g) T cell‐mediated cytotoxicity of *TP53^−/−^* PCI13 cells treated with isoprenaline in the presence or absence of a CXCL10‐neutralizing antibody. Differences were assessed using a mixed‐effects model with restricted maximum likelihood (REML) estimation; post hoc comparisons at specific T‐cell: tumor cell ratios were evaluated using Tukey's test. *P < 0.05. Error bars represent the standard error of the mean. Data show T cell‐mediated cytotoxicity, represented as a percentage of PCI‐13 cells expressing cleaved caspase‐3.

To identify the molecules potentially involved in this paracrine communication, we analyzed the CM from ISO‐treated *TP53* ^−/−^ PCI13 cell monocultures using Luminex assays (multiplex ELISA) with a panel of 30 cytokines (Table S4). CM was collected at different times after ISO treatment to evaluate the dynamics of cytokine release from p53‐deficient cancer cells. After 24 h, the CM from ISO‐treated cells showed significantly higher levels of IL‐6, IL‐8, CXCL10, TNFα, and IL‐1α compared to vehicle‐treated cells (Figure 3a). By day 4, CXCL10 and TNF‐α levels were notably higher in ISO‐treated cells compared to CM from vehicle‐treated cells (Figure 3b). These findings demonstrate that ISO prompts p53‐null cancer cells to release various inflammatory cytokines, which can directly or indirectly influence the activity of CD8+ T cells.

Based on these findings, we examined whether these cytokines were also expressed by oral keratinocytes from oral premalignant and OSCC samples from our p53‐null oral carcinogenesis mouse model. Considering that the cytokine release by OSCC was dependent on adrenergic stimulation, we initially assessed the abundance of sympathetic nerve fibers in mouse oral tissues by detecting a pan‐neuronal (*Calca*, *Cdh19*, *Gap43*, *Gfra1*, *Gpr37l1*, *Lgi4*, *Mag*, *Plp1*, *Pmp22*, *Uchl1*) and adrenergic (*Dbh*, *Slc18a2*, *Slc6a2*, *Th*) gene signature using spatial transcriptomics. The gene signatures were initially assessed for specificity by evaluating their expression within submucosal nerves and perivascular structures (Figure S4). Subsequently, the transcript densities of genes within each signature were measured exclusively in the epithelial layer and the subjacent lamina propria (Figure 3c; Figure S5). Adrenergic and pan‐neuronal signature gene transcripts were abundant in the epithelium and lamina propria layers of all analyzed samples, with no significant differences among animals with premalignant or malignant lesions, indicating a potentially ample source of adrenergic stimuli. This aligns with existing research showing that adrenergic nerve fibers are plentiful in the oral tissues of mice. This is especially true in the epithelial region of the oral mucosa, notably in mice undergoing chemically induced oral carcinogenesis, where sympathetic nerve remodeling occurs around oral lesions [35, 36, 37]. Next, we assessed whether the oral epithelium of these animals expressed the cytokines upregulated by isoprenaline in vitro (as shown in Figure 3a,b). Among these, *Cxcl10* was the most abundantly expressed in oral keratinocytes, detected in both OSCC tissues and in a single oral dysplasia tissue (Figure 3d; Figure S6a). *Tnf* and *Il6* were also expressed in oral keratinocytes from the same samples, overlapping spatially with regions of high *Cxcl10* expression, but at significantly lower levels (Figure S6a‐b). For all three cytokines, their expression was higher in OSCC than in premalignant lesions (Figure S6c). Expression of *Cxcl8 (Il8)* and *Il1a was* not evaluated, as a mouse homolog does not exist for the first and the second was not included in our Xenium panel. We also analyzed T cell density by measuring the transcript density of specific T cell genes. The transcript density of T cells (Cd3d, Cd3e, Cd3g) and CD8+ T cells (Cd8a, Cd8b1) generally coincided with the spatial distribution of Cxcl10 transcripts (Figure 3f). This also applied to transcripts linked to cytotoxic activity (*Gzma*, *Gzmb*, *Gzmk*, and *Prf1*) (Figure S7). Overall, these results indicate that oral premalignant and OSCC lesions from p53‐null animals also express *Cxcl10* (*Tnf* and *Il6* at lower levels), which is spatially linked to increased transcript density of T cell genes. To verify whether these findings could also be confirmed at the protein level and to evaluate if CXCL10 expression by oral keratinocytes correlates with ADRB2 expression, we performed multiplex immunofluorescence (mIF) on a new set of mouse oral mucosa samples obtained from 4NQO‐treated p53‐null mice. This analysis showed extensive expression of CXCL10 and ADRB2 proteins in oral epithelial keratinocytes (Pan‐CK+), with significant overlap (Figure 3f). Together, these findings suggest that β‐adrenergic signaling increases CXCL10 secretion in p53‐deficient OSCC cells, leading to increased T cell infiltration in the tissue microenvironment of both premalignant and malignant oral lesions.

Considering that Cxcl10 was the most abundant cytokine in our in vivo data, we decided to modulate it in vitro to evaluate its effect on isoprenaline‐induced enhancement of T‐cell‐mediated cancer cell killing. We conducted caspase‐3 cytotoxicity assays on TP53‐/‐ PCI13 cells with and without a neutralizing anti‐CXCL10 antibody. The results showed that blocking CXCL10 activity reversed the increase in T cell‐mediated cytotoxicity caused by isoprenaline, leading to a significantly lower cancer cell death rate, similar to that in untreated cells (Figure 3i).

### 2.4 Conditioned Media From p53‐Null Oral Cancer Cells Treated with Isoprenaline Alters CD8+ T Cell Transcription

**FIGURE 4:**
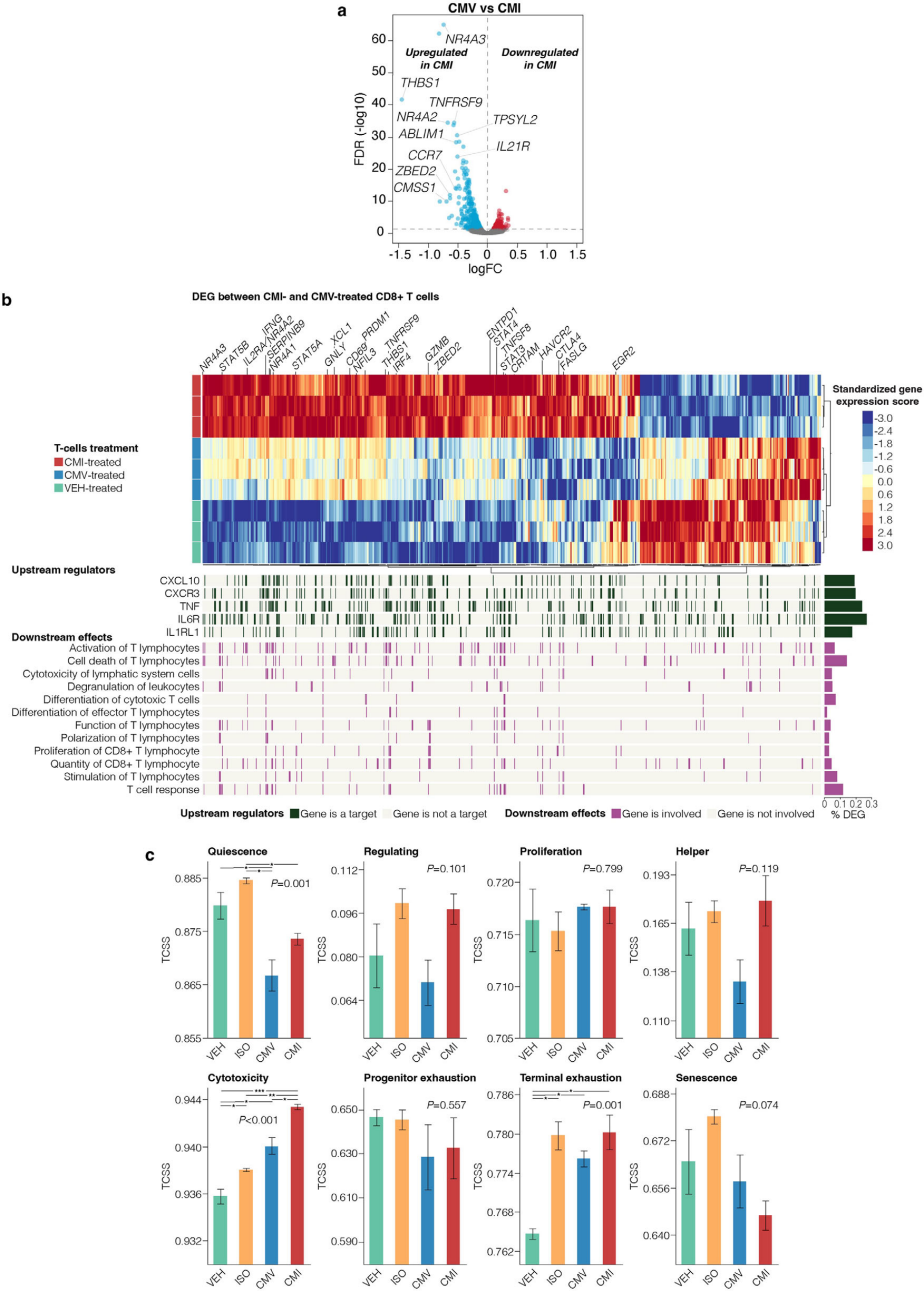
Analysis of the impact of factors derived from the *TP53^−/−^* PCI13 cell line, stimulated by isoprenaline, on the transcriptomic profile of CD8 T cells. a) The volcano plot shows the differentially expressed genes (DEG) between patient‐derived MART‐1‐specific T cells cultured in conditioned media from isoprenaline‐treated (CMI) or vehicle‐treated (CMV) *TP53^−/−^* PCI13 cells. Gene expression was assessed by bulk RNA sequencing. Differences in gene expression were assessed using the empirical Bayes quasi‐likelihood F‐test implemented in edgeR and the p‐values were corrected for multiple hypothesis testing (FDR) using the Benjamini–Hochberg method. b) Hierarchical clustering analysis considering the DEG between CMI‐, CMV‐treated, and control (vehicle‐treated) T cells (*upper heatmap*). Analysis in the Ingenuity Pathway Analysis (IPA) software revealed that CXCL10, CXCR3, TNF, IL6R, and IL1RL1 were significantly enriched as upstream regulators associated with the DEGs. DEGs related to these upstream regulators are shown as green bars in the *heatmap in the middle*. IPA analysis also indicated that the DEGs significantly enriched several downstream effects related to T cell function and differentiation. These DEGs are shown as magenta bars in the *heatmap at the bottom*. Transcriptional factors and other key genes to T cell function are highlighted in the heatmap. c) The bar plots illustrate the T Cell State Scores (TCSS)[17] for T cells subjected to CMI and CMV treatments, as well as T cells directly exposed to isoprenaline or a vehicle control. The TCSS was derived from the gene expression profiles of the T cells, with higher values indicating an enrichment of a particular T cell state. Differences were assessed using one‐way ANOVA followed by the Tukey‐Kramer HSD test for pairwise comparisons; *P < 0.05, **P < 0.001, ***P < 0.0001.

To better understand how adrenergic stimulation of p53‐deficient cancer cells affects the tumor microenvironment, we conducted transcriptome analysis on CD8+ T cells exposed to conditioned media from vehicle‐treated (CMV) and isoprenaline‐treated (CMI) *TP53* ^−/−^ PCI13 cells. The transcriptional profile of CD8+ T cells treated directly with either vehicle or isoprenaline was also assessed. The transcriptional profiles of CD8+ T cells treated with CMV and CMI were strikingly distinct, displaying 498 differentially expressed genes (DEG) (Figure 4a,b and Table S5). The transcriptional profile of CMV‐treated T cells was more similar to that of T cells subjected to a direct vehicle (control) treatment than to the CMI‐treated cells.

To determine whether the transcriptional differences correlate with the distinct cytokine profiles observed in the Luminex assay, we analyzed upstream regulators predicted to contribute to these transcriptional patterns. Among the significantly enriched *upstream regulators*, we identified CXCL10, along with its receptor CXCR3, TNF, the IL‐6 receptor (IL‐6R), and the IL‐1α receptor (IL1RL1). IL‐8 was not recognized as a significant upstream regulator (Figure 4b and Table S6). These findings suggest a potential influence of these cytokines on the transcriptional profile observed in CMI‐treated CD8 T cells. Next, we examined the predicted *downstream effects* associated with these DEGs. Various mechanisms related to T cell differentiation and cytotoxicity were predicted to be affected by the genes deregulated following CMI treatment (Figure 4b). A more detailed analysis showed that several DEGs between CMV‐ and CMI‐treated T cells were essential for CD8+ T cell functionality, including transcription factors (*NR4A1/2/3, STAT3, PRMD1, EGR2, NFIL3*), differentiation markers (*HAVCR2, ENTPD1, LAYN, CD69, BACH2*), mediators of T cell cytotoxicity (*GNLY, FASLG, IFITM1, GZMB, IFNG*), and co‐stimulatory molecules (*ICOS, TNFRSF9, SLAMF6*) (Figure 4b). These findings suggest that the cytokine expression profile induced by isoprenaline in p53‐deficient HNSCC cells leads to transcriptional changes in cytotoxic T cells that could alter their functional state.

To understand how these transcriptional changes influence functional states, we used a scoring tool (TCellSI, which calculates an enrichment score, or TCSS) to assess eight functional states based on the gene expression profiles of each T cell group [38]. Our results show that CMI‐treated T cells have a significantly higher Cytotoxicity score than T cells treated with CMV, as well as those given direct isoprenaline or vehicle treatments (Figure 4c). T cells exposed to CM from either vehicle‐treated or isoprenaline‐treated cancer cells exhibited lower *Quiescence* scores compared to those directly treated with isoprenaline or vehicle. This observation indicates that additional molecules derived from cancer cells within the conditioned media can induce T cells to exit quiescence, independent of isoprenaline. We also found a notable increase in the Terminal exhaustion signature in CMI‐ and isoprenaline‐treated T cells compared with CMV‐ and vehicle‐treated T cells, indicating a potential direct effect of isoprenaline on T cells. The CMI treatment prompts T cells to acquire transcriptional traits linked to both cytotoxic and terminally exhausted states. This exhausted effector profile exhibits reduced expression of genes related to T cell renewal, such as TCF7, while increasing the expression of renewal repressors like PRDM1, NFIL3, and the NR4A family members NR4A1, NR4A2, and NR4A3. At the same time, there is an increase in genes associated with cytotoxicity and effector functions, such as GZMB, SERPINB9, and IFNG. This suggests that, even with an exhausted transcriptional profile, these cells continue to exhibit characteristics of effector CD8 T cells, aligning with their increased cytotoxic activity seen in vitro (Figure 4b). These results may appear contradictory; however, previous research has shown that some terminally exhausted CD8+ T cells retain a cytotoxic phenotype and continue to contribute to tumor cell killing [39]. Notably, CD8+ T cells exposed to CMI do not exhibit the typical transcriptional profile of terminally exhausted T cells. Specifically, while *BATF* and *TOX* are usually elevated in such exhausted cells, our data show that these markers are lower in cells treated with CMI or CMV than in those treated with vehicle control. Despite other exhaustion markers, the reduced *BATF* suggests a non‐traditional, terminally effector‐exhausted state. Overall, these findings indicate that conditioned media from isoprenaline‐treated p53‐deficient HNSCC cells can activate, yet transiently, an effective cytotoxic state.

### 2.5 Blocking Adrenergic Signaling Suppresses Anti‐Tumor Immune Responses In Vivo

**FIGURE 5:**
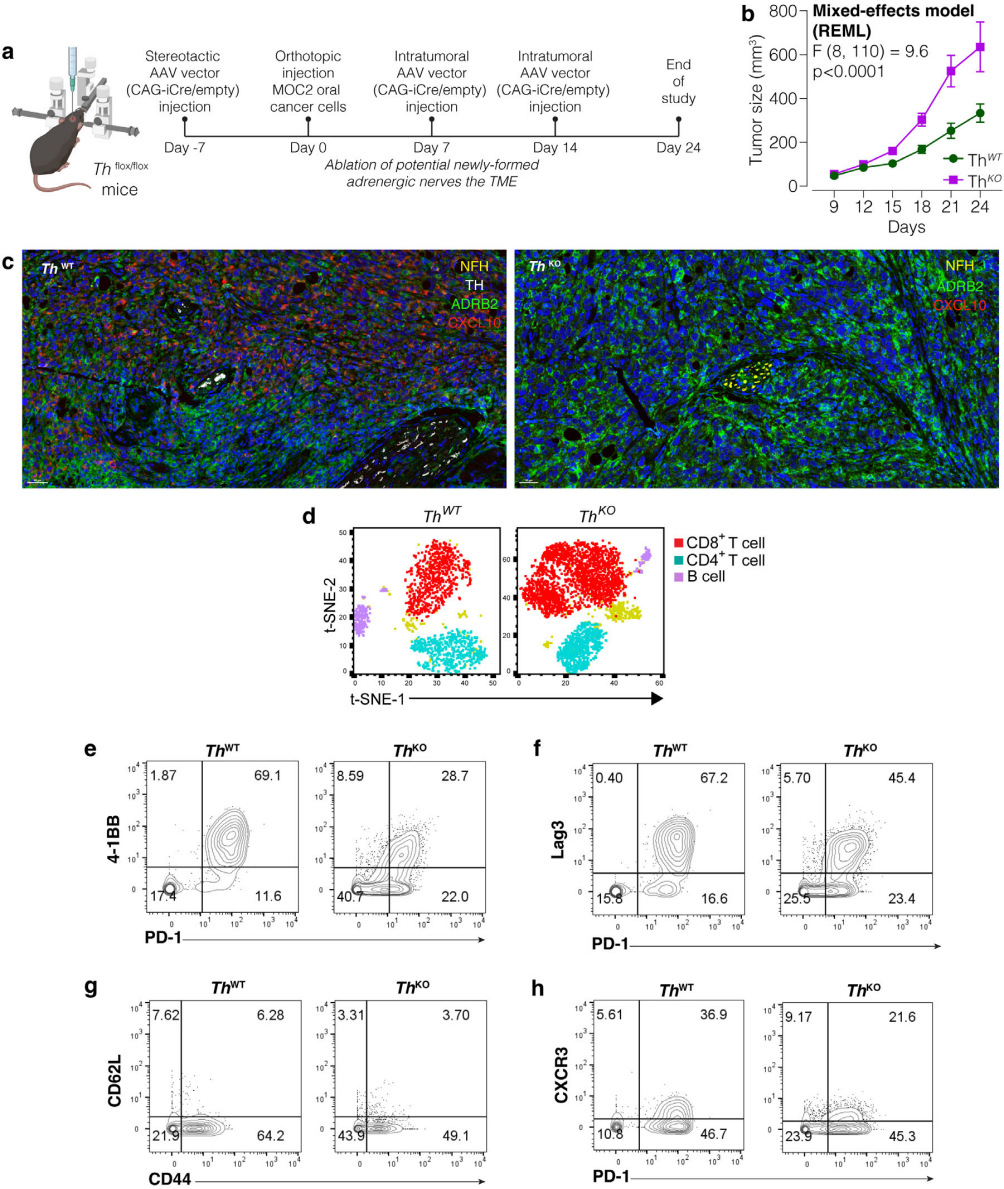
In vivo modulation of adrenergic signaling within an orthotopic oral cancer mouse model and its influence on tumor progression and CD8+ T cell phenotype. a) The figure illustrates the in vivo experimental approach employed to evaluate the effect of adrenergic signaling blockade on oral cancer growth and on the tumor‐associated T cells. MOC2 murine head and neck cancer cells were orthotopically injected in *Th* ^fl/fl^ (*adrenergic nerve‐specific gene*) mice (1×10^4^). AAV‐CAG‐iCre was injected into the tumor bed to ablate *Th* expression (*Th* ^KO^), resulting in tumoral sympathectomy, 7 days prior to cancer cell inoculation and subsequently at 7 and 14 days. The second and third injections were intended to ablate *Th* expression in newly formed adrenergic nerves within the tumor bed, which could potentially form after tumor initiation. Control animals (*Th* ^WT^) were injected with an empty viral vector. Animals were sacrificed 24 days following tumor cell injection. b) Tumor growth curve for *Th* ^KO^ and *Th* ^WT^ animals. n = 8 mice per condition, error bars represent the standard error of the mean. Differences were assessed using a mixed‐effects model with restricted maximum likelihood (REML) estimation; post hoc comparisons at specific time points were evaluated using Sidak's Multiple comparisons test. Tumor volume was significantly different between groups at day 18 (p=0.017), day 21 (p < 0.0001), and day 24 (p < 0.0001).  c) Multiplex immunofluorescence assessment of NFH, TH, ADRB2, and CXCL10 proteins in MOC2 orthotopic tumors derived from *Th* ^KO^ and *Th* ^WT^ animals. CXCL10 expression is elevated in *Th* ^WT^ animals but absent in *Th* ^KO^ ones. d) Mass cytometry (CyTOF) quantification of tumor‐infiltrating lymphocyte populations (gated on CD11b−CD45+) in MOC2 orthotopic tumors derived from *Th* ^KO^ and *Th* ^WT^ animals. Data are represented as t‐SNE maps overlaid with color‐coded populations. Quantification of CD8+ T cell populations was conducted based on the expression levels of 4‐1BB and PD‐1 (e), Lag3 and PD‐1 (f), CD62L and CD44 (g), and CXCR3 and PD‐1 (h). The numbers in each scatterplot denote the frequency of each intratumoral T cell population in *Th* ^KO^ and *Th* ^WT^ animals.

Our data so far indicate that adrenergic signaling in p53‐deficient HNSCC cells promotes paracrine activation of the anti‐tumor immune response. To verify whether this mechanism is also observed in vivo, we modulated the adrenergic signaling in an orthotopic mouse model of oral cancer. For this, we performed Cre‐mediated genetic ablation of adrenergic signaling within the tumor microenvironment in transgenic mice with the tyrosine hydroxylase (*Th*) gene flanked by loxP sites (Th^flox/flox^). The *Th* gene translates an enzyme that catalyzes the synthesis of catecholamines and is required for noradrenaline production by adrenergic neurons [40]. Th‐expressing innervation is encompassed by sympathetic postganglionic axons from the superior cervical ganglia (SCG) as well as maxillary and mandibular branches of the trigeminal nerve. We have previously shown that trigeminal sensory Th‐expressing neoneurites infiltrate the HNSCC TME as tumors progress [12]. MOC2 murine HNSCC cell lines were orthotopically injected at a dose of 10 000 cells per animal. These cells harbor a nonsense mutation in *Trp53*, leading to minimal to absent p53 protein activity [41]. Seven days before tumor injection, we stereotactically injected AAV‐CAG‐iCre (adeno‐associated virus expressing iCre recombinase under CAG promoter) or empty AAV vectors into the animals’ trigeminal ganglia, which provides innervation to the oral cavity via the maxillary and mandibular branches of the trigeminal nerve. Additional injections of AAV‐CAG‐iCre or a control AAV were administered at 7 and 14 days after tumor implantation, but now in the tumor bed. These interventions aimed to inhibit the adrenergic differentiation of other nerves within the TME (Figure 5a). A significantly higher tumor growth was observed in *Th* ^KO^ animals compared to *Th* ^WT^. We also examined these tumors using multiplex immunofluorescence (mIF), focusing on Cxcl10 expression. Notably, Cxcl10 expression was abundant in *Th* ^WT^ but completely absent in *Th* ^KO^ animals (Figure 5c). Next, we explored the immune phenotype linked to these tumors. The proportion of CD8+ T cells was marginally higher in *Th* ^KO^ (∼2.5%) animals compared to *Th* ^WT^ (∼1%) (Figure 5d). However, the magnitude of difference between groups was higher considering the different T cell phenotypes. The fraction of activated CD8+ T cells (PD‐1^+^4‐1BB^+^) was higher in *Th* ^WT^ animals than in *Th* ^KO^ (69.1% and 28.7%, respectively) (Figure 5e). Similarly, the fraction of exhausted CD8+ T cells (Lag‐3^+^PD‐1^+^) was higher in *Th* ^WT^ animals than in *Th* ^KO^ (67.2% and 45.4%, respectively) (Figure 5f). Additionally, in *Th* ^KO^ animals, there was a reduction in the frequency of effector memory CD8+ T cells (CD62L^−^CD44^+^) (49.1% and 64.2%, respectively) (Figure 5g) and a decrease in the frequency of CXCR3^+^PD‐1^+^ CD8+ T cells compared to *Th* ^WT^ animals (21.6% and 36.9%, respectively) (Figure 5h). Overall, these findings demonstrate that adrenergic signaling has a significant influence on tumor growth, Cxcl10 expression in tumors, and the phenotype of tumor‐associated CD8+ T cells.

### 2.6 P53 Loss Creates a Permissive Signaling State for β2‐Adrenergic Activation of CXCL10‐Associated TFs

**FIGURE 6:**
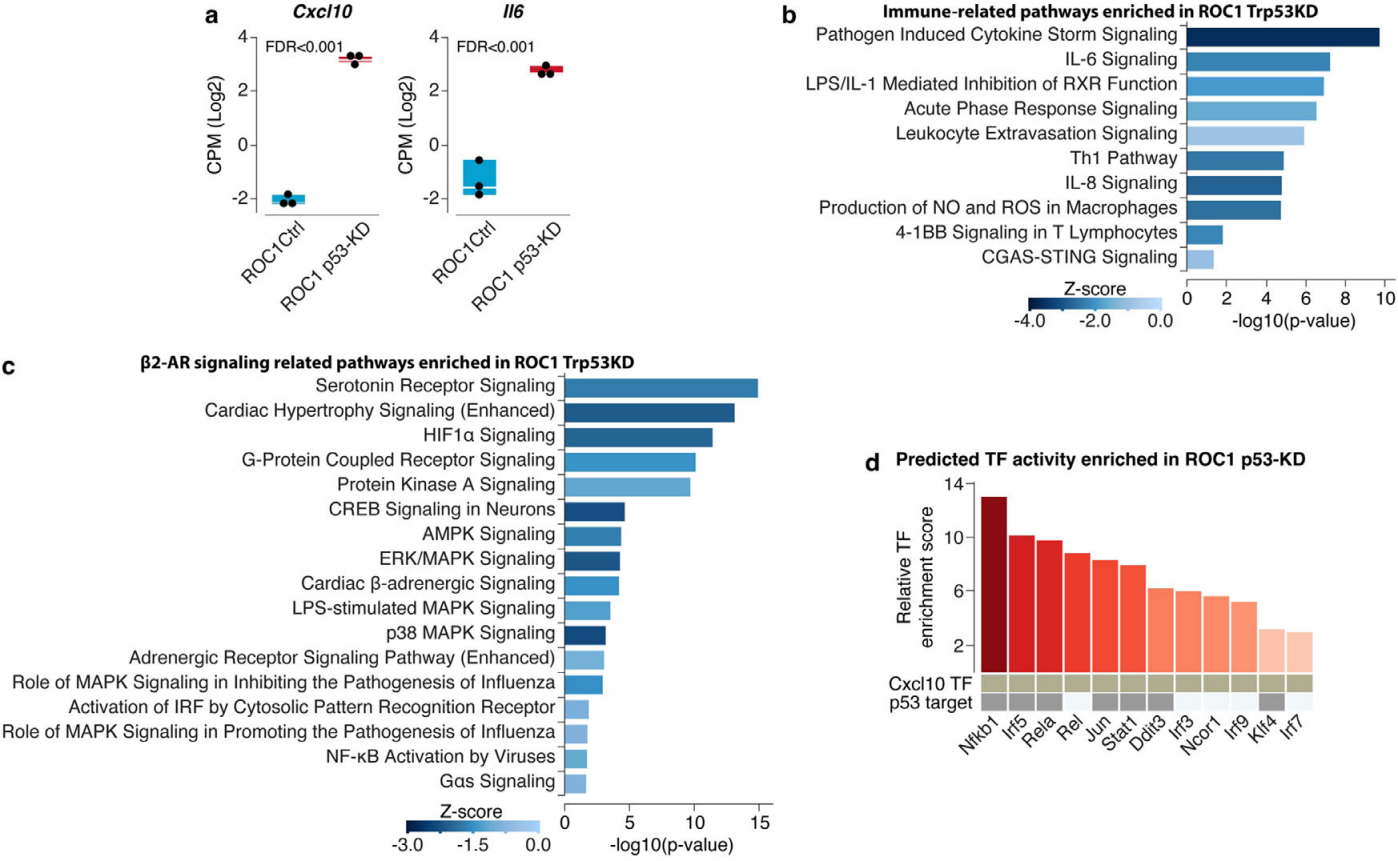
Loss of p53 rewires β2‐adrenergic downstream signaling and inflammatory transcriptional programs in ROC1 oral tumors. (a) Cxcl10 and Il6 expression in ROC1Ctrl and ROC1‐p53KD tumors, showing induction of Cxcl10 only upon p53 knockdown. Differences in gene expression were assessed using the empirical Bayes quasi‐likelihood F‐test implemented in edgeR and the p‐values were corrected for multiple hypothesis testing (FDR) using the Benjamini–Hochberg method. (b) Pathway analysis in IPA revealed immune‐related pathways enriched in ROC1‐p53KD tumors, suggesting a shift toward a pro‐inflammatory microenvironment. **(c)** β2‐adrenergic–associated signaling pathways are enriched in ROC1‐p53KD tumors, showing different activation of the canonical downstream signaling to ADRB2. **(d)** Transcription factor (TF) activity predicted by decoupleR highlights TFs enriched in ROC1‐p53KD tumors that regulate Cxcl10 expression and are modulated by p53. These data show that loss of p53 increases inflammatory TF programs and reveals β2‐adrenergic signaling outputs that specifically promote Cxcl10 induction.

To investigate the mechanisms underlying β2‐adrenergic signaling‐induced Cxcl10 expression specifically in the absence of p53, we revisited in silico data from our previous study, which showed that murine oral tumors derived from ROC1 cell lines display a significant immunological shift after Trp53‐knockdown (p53‐KD) [42]. Parental ROC1 cells (ROC1 Ctrl) develop immune‐cold tumors that exhibit limited CD8^+^ T‐cell infiltration and a cytokine environment that promotes regulatory T cells and M2‐like macrophages. Conversely, p53‐KD converts these tumors into highly immune‐infiltrated lesions with increased cytotoxic T cells and M1‐like macrophages. Supporting this immune shift, our in‐silico analysis of RNAseq data from ROC1Ctrl and ROC1‐p53KD tumors reveals that ROC1Ctrl tumors do not express *Cxcl10* and *Il6*, whereas ROC1‐p53KD tumors exhibit a significant increase in their expression in vivo (Figure 6A). To better understand the transcriptional differences between these tumors, we conducted pathway analysis (IPA), which revealed a significant enrichment of inflammatory pathways in ROC1‐p53KD tumors, predominantly centered around NF‐κB, MAPK, and interferon‐driven transcriptional programs (Figure 6B). These pathways are known to be transcriptionally suppressed by p53 [43, 44, 45], which aligns with p53's established inhibitory role in inflammatory genes. Notably, our observations also revealed that tumors with ROC1‐p53KD demonstrated a significant enrichment of pathways downstream of ADRB2 activation, including Gαs Signaling, Cardiac β‐adrenergic Signaling, GPCR Signaling, Protein Kinase A Signaling, CREB Signaling in Neurons, ERK/MAPK Signaling, and p38 MAPK Signaling (Figure 6C). These pathways embody canonical β2‐AR outputs capable of regulating transcription through cAMP–PKA‐, β‐arrestin‐, and MAPK‐dependent mechanisms [46, 47, 48, 49, 50, 51, 52]. The enrichment of these signatures suggests that the transcriptional outputs downstream of β2‐AR are differentially regulated in the absence of p53.

To determine which transcription factors (TF) connect these pathways to the increase in *Cxcl10* transcription, we inferred TF activity from the tumor RNAseq data (decoupleR package). Out of 561 tested regulons, 136 showed differential activity between ROC1Ctrl and ROC1‐p53KD tumors (Table S7). Twelve TFs showing increased activity in ROC1‐p53KD tumors were known regulators of Cxcl10 (Figure 6D). Among these, Nfkb1, Irf5, Rela, Jun, Stat1, Ddit3, and Klf4 have their TF activity influenced by p53 [43, 44, 45] and can also be affected by β2‐AR signaling [46, 47, 53]. These findings indicate that β‐adrenergic signaling stimulates Cxcl10 expression by activating an inflammatory TF network involving NF‐κB, STAT1, and IRF. In ROC1Ctrl tumors, this pathway may be suppressed by p53's inhibitory effects. Overall, the absence of p53 uncovers a β2‐adrenergic–responsive transcriptional pathway that specifically increases Cxcl10 expression, highlighting a common role for β2‐adrenergic signaling and p53‐dependent control of inflammatory responses.

## 3 Discussion

The development of effective immunotherapeutic strategies for HNSCC remains challenging, with conventional approaches often failing to overcome tumor‐intrinsic immune evasion mechanisms. While β‐adrenergic signaling has traditionally been associated with tumor progression and immunosuppression, recent report from our group have shown that the effect of β‐blockers in HNSCC is heterogeneous and the use of β‐blockers is associated with worse patient survival [20]. The findings presented in this study reveal a context‐dependent mechanism by which this pathway can unexpectedly enhance anti‐tumor immunity in p53‐deficient HNSCC. This discovery provides mechanistic insight into the complex neuro‐immune interactions that govern cancer immunosurveillance and identifies novel therapeutic opportunities for patients with p53‐mutant tumors.

Our findings reveal that β‐adrenergic stimulation can override immune evasion in p53‐deficient HNSCC by unleashing CXCL10‐driven paracrine activation of cytotoxic T cells. Strikingly, CXCL10 emerges as a pivotal hub of this neuro‐immune circuitry: neutralizing CXCL10 completely abolishes the isoprenaline‐triggered boost in T cell‐mediated tumor destruction, underscoring its indispensable role. Complementary loss‐of‐function experiments in tyrosine hydroxylase knockout mice further establish that adrenergic innervation is absolutely required for tumor CXCL10 production and robust activation of intratumoral T cells. These findings align with the established role of CXCL10 in promoting anti‐tumor immunity, where it serves both as a chemoattractant for CXCR3‐expressing immune effector cells and as a functional modulator of T cell responses[25]. The identification of CXCL10 as a significant upstream regulator in this transcriptional program further supports its central role in mediating the observed immune enhancement. The profound loss of both CXCL10 and CD8+ T cell activation markers in ThKO tumors highlights a direct mechanistic bridge between neural signals and immune effector function.

Notably, this pathway creates an interesting duality as CD8+ T cells exposed to CXCL10 simultaneously show increased activation and exhaustion markers, indicating ongoing, strong antigen engagement. Yet, the sustained effector state within this context may offer new therapeutic levers, as it holds promise for circumventing the classic hurdle of T cell exhaustion in immunotherapy.

Based on T cell phenotyping from human in vitro RNA sequencing data, we found that CD8^+^ T cells treated with conditioned media from isoprenaline‐treated (CMI) p53‐null HNSCC cells show enrichment of exhaustion‐associated genes and increased expression of cytotoxic genes. This phenotype aligns with the “effector‐like exhausted” dysfunctional CD8^+^ T cell profile, characterized by co‐expression of activation markers (4‐1BB, ICOS, GZMB, IFNG), inhibitory receptors (PD‐1, CTLA‐4, TIM‐3), and dysregulation of transcription factors such as NR4A1‐3, BATF, and PRDM1, all differentially regulated by CMI treatment [59, 60, 61]. Since our phenotyping relied on bulk RNA sequencing, our sample likely contains a mixture of exhausted and cytotoxic T cells, which can be more precisely distinguished with single‐cell RNA sequencing. Nonetheless, flow cytometry analysis of in vivo tissue also revealed a similar heterogenous CD8^+^ T‐cell phenotype with both activation and exhaustion features in tumors from ThWT animals. Together, these in vitro and in vivo data suggest that CD8 T cells exposed to p53‐deficient HNSCC tumors exhibit a phenotype characteristic of antigen‐experienced CD8^+^ T cells, combining exhaustion features with maintained cytotoxic potential. Similar cells have been described in melanoma and myeloma [39, 59, 60].

A comparable population of effector‐like exhausted CD8 T cells with high 4‐1BB (also known as CD137 or TNFRSF9) expression was recently identified in a humanized tumor model [62]. These cells showed effective antitumor activity in an in vivo adoptive transfer model of autologous humanized tumors. Interestingly, similar effector‐like exhausted CD8 T cells have also been observed in HNSCC, where their presence correlates with a positive response to anti‐PD‐1 and anti‐PD‐L1 immunotherapy [63, 64].

Importantly, the β‐adrenergic‐induced CXCL10 response depends critically on p53 status, with p53‐deficient cells demonstrating enhanced chemokine production compared to p53‐wild‐type cells. This finding suggests that p53 loss creates a permissive environment for adrenergic‐stimulated inflammatory cytokine production, potentially through the loss of p53's regulatory role in inflammatory responses and cellular homeostasis [6, 65]. p53 typically functions as a brake on excessive inflammatory signaling, and its loss may unleash enhanced cytokine production in response to stress stimuli such as adrenergic activation [65].

Beta‐adrenergic agonism enhances cyclic AMP levels, thereby activating PKA and other downstream signaling pathways that ultimately regulate NF‐κB and MAPK signaling. NF‐κB, for example, promotes the transcription of cytokines such as CXCL10, TNF‐α, and IL‐6, which are well recognized as canonical NF‐κB targets [47, 66, 67]. It has been demonstrated that the β2‐AR antagonist ICI118,551 inhibits the p38 and NF‐κB pathways in TP53 mutant HNSCC cell lines (UMSCC103 and CAL‐33) [17]. Conversely, in models possessing functional p53, p53 serves as a negative regulator of NF‐κB transcription by sequestering co‐activators such as CBP/p300, inhibiting NF‐κB DNA binding, or augmenting IκBα expression [68]. Our results indicate that the loss of p53 fundamentally alters intracellular signaling networks, creating an environment in which β2‐adrenergic signaling promotes pro‐inflammatory transcriptional responses. Therefore, in cells with impaired p53 function or TP53 mutations, beta‐adrenergic signaling might enhance cytokine expression. Additionally, mutant p53 proteins can gain oncogenic functions that actively promote inflammatory signaling pathways [69, 70]. The discovery that β‐adrenergic agonists can enhance anti‐tumor immunity specifically in p53‐deficient HNSCC challenges conventional understanding of stress‐cancer relationships. While chronic stress and β‐adrenergic signaling typically promote tumor progression, our findings reveal that cellular context—particularly p53 status—determines the ultimate outcome. This suggests potential therapeutic utility for β‐adrenergic modulators in specific cancer subtypes, particularly those harboring p53 deficiency.

Our study uncovers a compelling new paradigm: β‐adrenergic signaling empowers anti‐tumor immunity in p53‐deficient HNSCC by engaging a CXCL10‐driven axis of T cell activation. This work not only establishes CXCL10 as a pivotal molecular bridge linking neural signaling, tumor suppressor loss, and immune surveillance but also delivers a concrete, targetable pathway for innovative therapeutic intervention. Yet, this discovery prompts provocative new questions. The precise intracellular circuitry through which β‐adrenergic inputs upregulate CXCL10 in p53‐deficient tumor cells remains to be elucidated; dissecting these molecular events could yield key targets for intervention and clarify stress‐related tumor biology. The durable impact of sustained CXCL10 signaling, both in potentiating CD8+ T cell responses and in shaping patterns of immune resistance over time, awaits deeper analysis.

A broader investigation is necessary to determine whether this neuro‐immune mechanism operates in other high‐frequency TP53 mutant cancers and to validate its relevance in patient‐derived tissues. Equally critical is the urgent need for biomarkers that can predict which patients are most likely to benefit from precision neuro‐immunomodulatory therapy. Together, these lines of inquiry will be essential for translating our findings into rational, personalized treatment approaches and for realizing the full therapeutic potential of the neuro‐immune interface in HNSCC.

The therapeutic potential of β‐adrenergic modulation in TP53‐deficient HNSCC must be carefully evaluated considering the established tumor‐promoting effects linked to β2‐AR signaling. Previous studies show that activation of β2‐AR can promote epithelial‐mesenchymal transition (EMT), cell migration, and invasion in oral squamous cell carcinoma, with overexpression of β2‐AR associated with aggressive clinical features and poor prognosis [19, 71, 72, 73]. Similarly, stress‐induced β‐adrenergic activation has been linked to tumor progression and metastasis in head and neck cancers, as well as other solid tumors [16, 74, 75, 76, 77]. These inherent tumor effects present a significant clinical challenge that must be carefully addressed in developing therapies. However, our findings reveal a key context‐dependent feature of the β2‐AR pathway: in tumors lacking p53, it shifts from promoting tumor growth to increasing CXCL10 production and recruiting T cells. This suggests that the immunostimulatory and tumor‐promoting effects of β‐adrenergic signaling are influenced by the tumor's genetic makeup and immune environment. Recognizing this distinction is crucial for accurately classifying patients and tumors, yet it remains feasible within clinical practice.

To harness the immune‐stimulatory potential of β‐adrenergic signaling while minimizing the risk of tumor promotion, we propose several complementary strategies. First, pro‐adrenergic treatments should be limited to tumors with confirmed TP53 loss‐of‐function mutations and evidence of immune exclusion, such as low baseline CD8+ T cell infiltration and low CXCL10 expression. This molecular gating ensures that β2‐AR modulation is applied only in contexts likely to trigger the immune‐stimulatory transcriptional reprogramming observed in our study. Although β2‐AR is expressed in nearly all head and neck tumors [78], β‐adrenergic signaling depends not only on receptor levels but also on downstream regulatory molecules like β‐arrestins, GRKs, and PDE4, which influence how β2‐AR couples with G proteins and can affect its downstream signaling pathways [52, 79, 80, 81]. Since changes in these molecules have been seen in HNSCC [82, 83, 84, 85, 86], a deeper understanding of β‐adrenergic signaling in HNSCC is needed to better identify tumors that could respond to β‐adrenergic stimulation as anticipated. Second, administering β2‐agonists directly into the tumor or nearby regions, rather than systemically, would maximize CXCL10 production at the tumor site while reducing systemic exposure and the potential for off‐target tumor‐promoting effects at distant metastatic sites. Alternatively, directly injecting CXCL10 into tumors to target the CXCL10‐CXCR3 axis offers another method for immune activation that avoids directly stimulating β2‐AR. Third, controlling β‐adrenergic stimulation within specific therapeutic windows, synchronized with immune checkpoint inhibitor treatments, could improve T cell recruitment and activation while avoiding prolonged β2‐AR stimulation that might promote tumor growth. Clinical and experimental data show that CXCL10 expression correlates with better responses to immunotherapy in various cancers [87, 88, 89, 90, 91, 92], and our findings provide a mechanistic basis for combining controlled β2‐adrenergic stimulation with immunotherapy in TP53‐deficient HNSCC.

In conclusion, although β‐adrenergic signaling has dual, context‐dependent effects in HNSCC, careful molecular stratification, targeted local delivery methods, and strategic combinations with immunotherapy offer rational approaches to leverage its immune‐boosting potential while reducing tumor‐promoting risks. Future clinical efforts should focus on early‐phase trials involving molecularly selected TP53‐deficient, immune‐excluded HNSCC patients, with close monitoring of immune activation and tumor progression.

## 4 Methods

### 4.1 4‐NQO Oral Carcinogenesis Mouse Model

To induce oral squamous cell carcinoma (OSCC), we employed the well‐established 4‐nitroquinoline‐1‐oxide (4NQO) chemical carcinogenesis model, which mimics the progressive histopathological stages of human oral cancer, from hyperplasia to dysplasia and invasive squamous cell carcinoma. All animal procedures were approved by the Institutional Animal Care and Use Committee (IACUC) of The University of Texas MD Anderson Cancer Center. Male and female p53‐null C57BL/6J mice aged 6–8 weeks were housed under standard conditions with free access to food and water. 4NQO (Sigma‐Aldrich) was freshly prepared at 75 µg/mL in drinking water and administered freely for 12 weeks. After exposure, all mice were returned to regular water and monitored for an additional 8–12 weeks to allow for lesion development and progression. Weekly surveillance included checking for signs of morbidity, weight loss, and visible oral lesions. The tongue and oral mucosa were visually examined weekly. Lesions were classified based on size, ulceration, and location.

At the endpoint, mice were euthanized, and oral tissues were collected. Tongues were split longitudinally, fixed in 10% neutral‐buffered formalin for histology and spatial transcriptomics. An oral pathologist reviewed H&E‐stained sections to classify each oral lesion. Lesions were considered premalignant if clinical lesions showed dysplastic changes in the oral epithelium or as oral squamous cell carcinoma when they invaded the lamina propria. Dysplastic lesions were classified as mild if limited to the basal and suprabasal layers, or as moderate/severe if they involved middle and upper epithelial layers, showed drop‐shaped or bulbous projections, or displayed architectural disorganization and maturation loss.

### 4.2 Spatial Transcriptome

FFPE tissue samples, as part of a tissue microarray, were sectioned at a thickness of 5 µm and mounted onto Xenium‐compatible glass slides (10× Genomics). To facilitate cell and nuclear segmentation while maintaining tissue morphology, tissue sections were incubated with the Xenium Multi‐Tissue Morphology Stain Mix (PN‐2000991). This Mix comprised antibodies for cell boundary segmentation targeting ATP1A1, CD45, and E‐cadherin; for intracellular protein staining, targeting α‐SMA and vimentin; a fluorescent 18S RNA probe for cytoplasmic labeling; and DAPI for nuclear labeling and segmentation. A custom‐designed mouse panel targeting 479 genes was used, selected for cell type resolution and functional diversity. The Xenium In Situ platform (10× Genomics) was used for spatial transcriptomic profiling, performed according to the manufacturer's protocol. Raw image files were processed using the Xenium Onboard software to generate spot‐level transcript calls with subcellular resolution. Raw data, including transcript counts, cell segmentation, and spatial coordinates, were exported in HDF5 and CSV formats using Xenium Explorer. Quality control metrics included total transcript counts per cell, number of genes per cell, and spatial distribution uniformity. Cells with low transcript abundance or poor morphology were excluded from downstream analyses. Data were imported into R using Seurat (v5.0.1). Gene expression data were normalized using total transcript count per area‐normalized for spatial correction. Dimensionality reduction was performed, and principal component analysis (PCA) was conducted on the scaled gene expression data, utilizing highly variable features.

### 4.3 Cell Culture

PCI‐13 is a human cell line derived from an oral cavity squamous cell carcinoma tumor. It carries a TP53 frameshift deletion (p.N29fs) and a missense mutation (p.P72R) and does not express p53 protein, neither at baseline nor under stress [93]. In this study, we used PCI‐13 cells transduced with a vector encoding TP53 to induce p53 re‐expression or with an empty control vector. The TP53 mutation status and the restoration of p53 function in these cells were verified, with the results published elsewhere [28]. Dr. Jeffrey Myers kindly provided these cell lines.

This cell was then transduced with a T‐cell receptor specific for HLA‐A2, enabling it to present the peptide melanoma antigen (MART‐1) recognized by T cells and be recognized by MART‐1‐specific T cells. The cell line was cultured in Dulbecco's Modified Eagle's Medium (DMEM) supplemented with 10% fetal bovine serum (FBS), 1x non‐essential amino acids (NEAA), 1x pyruvate, 2 mM glutamine, and 50 U/mL penicillin/streptomycin. It was The MART‐1‐specific T cells were kindly provided by Dr. Patrick Hwu (Moffitt Cancer Center). Murine cell lines: MOC2 mouse HNSCC cells were obtained from the laboratory of Jeffry N. Myers. All tumor cell lines were regularly tested for mycoplasma contamination using the MycoAlert Mycoplasma Detection Kit (Lonza, cat #LTOz‐318) and verified as negative before use for various assays and studies. All cells were maintained at 37°C in an atmosphere containing 5% CO_2_.

### 4.4 Drugs Targeting Adrenergic Receptors

Atenolol (#A7655) (β1/β2‐adrenergic receptor antagonist), ICI 118,551 hydrochloride (#I127) (β2‐adrenergic receptor inverse agonist), isoproterenol hydrochloride (isoprenaline; #1351005) (β1/β2/β3‐adrenergic receptor agonist), and norepinephrine (#A7257) (Pan‐adrenergic receptor agonist) were purchased from Sigma‐Aldrich. All reagents were prepared using double‐distilled water with 0.22 µm filtration, and the final concentration was determined by the MTT (3(4,5‐dimethyl‐thiazol‐2‐yl)‐2,5‐diphenyltetrazolium bromide) assay.

### 4.5 Coculture and Cleaved Caspase‐3 Cytotoxicity Assay

Human HNSCC (PCI‐13*TP53* ^+/+^HLA‐A2 and PCI‐13*TP53* ^−/−^HLA‐A2) cells were pretreated with atenolol, carvedilol, ICI 118,551 hydrocholoride, isoprenaline, norepinephrine, xylazine, and yohimbine. The supernatants of the pretreated cells were collected after 24 and 48 h and stored at −80°C. The cells were then trypsinized, washed with PBS, and incubated at 37°C for 15 min after staining with DDAO (Thermo Fisher Scientific, #D6488). After incubation, the excess DDAO was washed and pulsed with the human MART‐1‐specific peptide (MBL International Corp, #SP0009) at a concentration of 1 µmol/L and incubated at 37°C for 1 h. The peptide was then removed from the cells. The excess MART‐1 peptide was removed after washing with PBS, and the cells were resuspended with the previously collected cell supernatant. Human HNSCC cells (2 × 10^3^) were plated in 96‐well plates and cocultured with human MART‐ specific patient‐derived T cells in various ratios (0 [T cells]:1 [HNSCC cells], no T cells; 0.5:1, 1000 T cells; 1:1, 2000 T cells; 2:1, 4000 T cells) and incubated for 1 h at 37°C. The coculture cells were washed, trypsinized, and resuspended with 1% paraformaldehyde and incubated for 20 min at room temperature. The cells were then incubated on ice with Cytofix (BD Biosciences, #554655) for 20 min in a dark place. The cells were washed with 1× perm buffer and incubated with caspase‐3‐ PE (BD Biosciences, #550914) antibody for 30 min in the dark at room temperature. After incubation, cells were resuspended with FACS buffer and cleaved caspase‐3. T‐cell cytotoxicity was analyzed by flow cytometry. Murine HNSCC and pancreatic KPC cells were treated with atenolol, denopamine, fenoterol, ICI 118,551 hydrocholoride, isoprenaline, and propranolol. Murine cells were pulsed with gp100/pmel17 [human equivalent of MART‐1] (Miltenyi Biotech #130‐094‐449) and co‐cultured with pMEL‐1 mouse‐derived CD8a T cells. The assay for cleaved caspase‐3 cytotoxicity was performed as described previously [94]. Each experiment was performed in triplicate, and data were presented as a percentage of T‐cell cytotoxicity. For CXCL10 neutralization, cultures were treated with 2 µg/mL of mouse anti‐human CXCL10 neutralizing monoclonal antibody (R&D Systems, Cat# MAB266) or an isotype control for 24 h.

### 4.6 IncuCyte Immune Cell Killing Assay

Human HNSCC cells (PCI‐13*TP53* ^+/+^HLA‐A2 and PCI‐13*TP53* ^−/−^HLA‐A2) were pretreated with isoprenaline, and cell supernatants were collected after 24 and 48 h and stored at −80°C. HNSCC cells were stained with IncuCyte CytoLight Rapid Red reagent (Sartorius, #4706) according to the manufacturer's instructions. Cells were then pulsed with human MART‐1‐specific peptide for 1 h and resuspended with supernatant as previously described. 3 × 10^3^ HNSCC cells were plated out in 96‐well plates and allowed to attach at 37°C for 2 h. After 2 h, human MART‐1 specific T cells were added to HNSCC cells according to the ratio of experimental conditions and incubated with IncuCyte Caspase‐3/7 Green Apoptosis Reagent (1:3500 dilutions; Sartorius, #4440) to determine the number of apoptotic cells. Similarly, 3.5 × 10^3^ murine HNSCC cells were pulsed with gp100/pmel17 and cocultured with pMEL‐1 mouse‐derived CD8a T cells. Real‐time images were acquired at 1‐h intervals, and data were analyzed using IncuCyte 2022B Rev2 software. Each experiment was performed in triplicate, and data were presented as percentage of T‐cell cytotoxicity.

### 4.7 Impedance‐Based Immune Cell Killing Assay

Cancer cells (PCI‐13p53WT HLA A2+; PCI‐13pBABE HLA A2+) were cultured at 37°C incubator with 5% CO2 in media containing DMEM with high glucose and pyruvate, 10% FBS, P/S, 1x vitamin and 1x NAA. 10 µM isoprenaline‐treated conditioned media (CM) was collected from 24‐ and 48‐h cultures of cancer cells in ImmunoCult‐XF T Cell Exp Medium (cat#10981, STEMCELL Tech), and then mixed with both supernatant and filtered. Hu PB CD8+ T cells (cat # 200‐0164, STEMCELL tech) were cultured according to the vendor protocol. Cells were grown in ImmunoCult‐XF T Cell Exp Medium with 10 ng/ml of Human Recombinant IL‐2 (Cat# 78036, STEMCELL tech). These cells were activated using 25 µl/ml of ImmunoCult Hu CD3/CD28 Act (Cat# 10991, STEMCELL tech) for 24 h from day 5 of culture. The activated Hu PB CD8+ T cells were then cocultured with cancer cells for the cytolysis assay.

Impedance‐based cell analysis platforms for real‐time (Impedance Module, AxIS Z software from Maestro Edge, Axion Biosystems) were used for the cytolysis assay. The Electrical Impedance plate (96‐well, Cyto View‐Z, Axion Biosystems) was coated with 1 µg/ml fibronectin (cat# 11051407001, Sigma‐Aldrich) for at least 1 h in a 37°C incubator. The fibronectin was then replaced with 100 µl of cancer cell culture media. The AxIS Z was set up for reading, and a baseline reading was taken. Data acquisition and analysis were performed using Axion Biosystems' AxIS Z software. Cancer cells (10 000 cells for PCI‐13p53WT HLA A+ and PCI‐13pBABE HLA A+, and 15 000 cells for all KO clones) were seeded in cancer cell media, with four replicates per treatment per cell line, and sterile water was added around the edge of the plate to maintain humidity and initiate recording. After 19 h of culture in Maestro Edge, the cancer cell media was replaced with or without 10 µM Isoprenaline, in ImmunoCult‐XF T Cell Expansion Medium containing 10 ng/ml of Human Recombinant IL‐2. Recording continued for the next 8 h. Then, 100 000 T cells, activated 24 h prior with ImmunoCult Hu CD3/CD28 or as inactive Hu PB CD8+ T cells, were added per well according to the assay plate map. The recording of the cancer and T cell coculture was continued for another 18 h.

### 4.8 MDM2 Transduction of Human HNSCC Cells

To induce stable ectopic expression of human MDM2 protein, *TP53* ^+/+^ PCI‐13 cells were transduced with a EF1A‐driven human MDM2 lentiviral expression vector (VectorBuilder, Cat# VB200124‐1076jqe) encoding blasticidin resistance under the mPGK promoter (Figure S3a). Cells were cultured in high‐glucose Dulbecco's Modified Eagle Medium supplemented with 10% FBS, 1× NEAA, 1× pyruvate, 2 mM glutamine, and 50 U/mL penicillin/streptomycin. The transduction protocol was performed according to the manufacturer's instructions (VectorBuilder Inc). Briefly, 3 × 10^5^ PCI‐13*TP53* ^WT^ cells were infected with 30 µL of virus (1 × 10^8^ TU/mL) containing 1 µL of polybrene (5 mg/mL). After 18 h, the media were replaced with fresh medium containing 10 µg/mL blasticidin for selection. Stable cell populations emerged within 10 days and were expanded. hMDM2 overexpression was confirmed under mild hypothermic (32.5°C) stress conditions at 24 h and 40 h by Western blot using the anti‐MDM2 (SMp14, Santa Cruz # Sc‐965) and anti‐actin (Sigma Aldrich #A2066) primary antibodies (Figure S3b). T‐cell cytotoxicity was determined using the cleaved caspase‐3 assay, as described above.

### 4.9 In Silico Data and Analysis

To assess the expression of ADRB2 in cancer, RNA expression data from solid tumors from TCGA were downloaded (March 2023‐“EBPlusPlusAdjustPANCAN_IlluminaHiSeq_RNASeqV2.geneExp.tsv”) from the NCI Genomic Data Commons website (https://gdc.cancer.gov/about‐data/publications/pancanatlas) [95, 96]. Normalized gene expression data were log_2_‐transformed before analysis. Only data from primary lesions (n = 9636 samples) and solid tumors (31 tumor types) were considered in the analyses. The tumor types and their respective number of samples are: rectum adenocarcinoma (READ) n = 160, colon adenocarcinoma (COAD) n = 449, adrenocortical carcinoma (ACC) n = 79, ovarian serous cystadenocarcinoma (OV) n = 305, uterine corpus endometrial carcinoma (UCEC) n = 532, cholangiocarcinoma (CHOL) n = 36, uterine carcinosarcoma (UCS) n = 57, stomach adenocarcinoma (STAD) n = 415, thyroid carcinoma (THCA) n = 505, testicular germ cell tumors (TGCT) n = 134, pheochromocytoma and paraganglioma (PCPG) n = 179, skin cutaneous melanoma (SKCM) n = 103, breast invasive carcinoma (BRCA) n = 1097, mesothelioma (MESO) n = 87,pPancreatic adenocarcinoma (PAAD) n = 178, lymphoid neoplasm diffuse large B‐cell lymphoma (DLBC) n = 48, glioblastoma multiforme (GBM) n = 155, bladder urothelial carcinoma (BLCA) n = 408, kidney renal clear cell carcinoma (KIRC) n = 533, esophageal carcinoma (ESCA) n = 184, thymoma (THYM) n = 120, lung squamous cell carcinoma (LUSC) n = 502, kidney renal papillary cell carcinoma (KIRP) n = 290, sarcoma (SARC) n = 259, lung adenocarcinoma (LUAD) n = 515, brain lower grade glioma (LGG) n = 515, liver hepatocellular carcinoma (LIHC) n = 371, cervical squamous cell carcinoma and endocervical adenocarcinoma (CESC) n = 305, kidney chromophobe (KICH) n = 66, uveal melanoma (UVM) n = 80, head and neck squamous cell carcinoma (HNSC) n = 520, and prostate adenocarcinoma (PRAD) n = 497 (https://www.cancer.gov/ccg/research/genome‐sequencing/tcga/studied‐cancers).

To assess the expression of ADRB1, ADRB2, and ADRB3 accross various cell types within the oral tissue, single‐cell RNA‐seq data from normal oral mucosa, oral premalignant lesions, and oral squamous cell carcinoma were sourced from the GSE181919 dataset. The analyss were conducted using the R package Seurat (v5.3.1).

### 4.10 Conditioned Media Experiment

Human HNSCC cells (PCI‐13*TP53* ^+/+^HLA‐A2 and PCI‐13*TP53* ^−/−^HLA‐A2) were cultured in complete medium. Cells and supernatants treated with isoprenaline or vehicle were collected after 24 and 48 h and stored at ‐80°C. The untreated human MART‐1‐specific T cells were cocultured with HNSCC cells, and these cocultured cells were treated with HNSCC cell supernatants from isoprenaline or vehicle treatment. T‐cell cytotoxicity was performed using the cleaved caspase‐3 assay as described above [94]. Each experiment was performed in triplicate, and data were presented as percentage of T‐cell cytotoxicity.

### 4.11 Multiplex Cytokine Analysis

Human HNSCC cells (PCI‐13*TP53* ^−/−^HLA‐A2) and patient‐derived MART‐1sspecific T cells, alone or in coculture with HNSCC: T cells, were treated with isoprenaline or vehicle. After 3 h of treatment, the cell secretome was collected, and cytokines, chemokines, or growth factors were analyzed using the Human XL Cytokine Luminex Performance Assay 45‐plex Fixed Panel [97]. The assay was performed according to the manufacturer's instructions. Briefly, the wells of the 1.2‐µm filter membrane 96‐well microtiter plates were prewetted with assay buffer. Next, 25 µL of the sample, standard, and quality control preparations were added to the relevant wells and incubated with the premixed microspheres for 2 h on an orbital plate shaker at room temperature. The plates were washed twice with assay wash buffer, and 25 µL of biotinylated detector antibody was added per well. Samples were incubated for 1 h at room temperature on the plate shaker. Without washing, 25 µL/well of streptavidin–phycoerythrin solution was added, and the plates were incubated for an additional 30 min at room temperature on a plate shaker, protected from direct light. The instrument was set to collect at least 50 beads per analyte. Raw data were measured as mean fluorescence intensity.

### 4.12 T‐Cell RNA Sequencing Analysis

Human HNSCC cell (PCI‐13*TP53* ^−/−^HLA‐A2) and patient‐derived MART‐1‐specific T‐cells were cocultured and treated with isoprenaline or vehicle. After 4 h of treatment, T cells were harvested and gene expression was assessed by RNA sequencing. Low‐input RNA libraries compatible with Illumina were prepared using the Smart‐Seq V4 Ultra Low Input RNA (Clontech) and KAPA HyperPlus library preparation kits. In brief, full‐length, double‐stranded cDNA was generated from 10 ng total RNA using Clontech's SMART (switching mechanism at 5′ end of RNA template) technology. The full‐length double‐stranded cDNA was amplified by eight cycles of long‐distance PCR, then purified using AMPure Beads (Agencourt). Following bead elution, the cDNA was evaluated for size distribution and quantity using the 4200 TapeStation High Sensitivity DNA Kit (Agilent Technologies) and the Qubit dsDNA HS Assay Kit (Thermo Fisher), respectively. The cDNA was enzymatically fragmented, and 5 ng of the fragmented cDNA was used to generate Illumina‐compatible libraries using the KAPA HyperPlus Library Preparation kit. The KAPA libraries were purified and enriched with eight cycles of PCR to create the final cDNA library. The libraries were quantified using the Qubit dsDNA HS Assay (Thermo Fisher), then multiplexed with 12–16 libraries per pool. The pooled libraries were quantified by quantitative PCR using the KAPA Library Quantification Kit (KAPA Biosystems) and assessed for size distribution using the 4200 TapeStation (Agilent Technologies). The libraries were then sequenced, one pool per lane, on an Illumina HiSeq 4000 sequencer using the 76‐bp paired‐end format. The raw FASTQ reads were mapped to the human genome (hg38) and transcriptome (GENCODE v23) using HiSAT2 (v2.1.0) [98]. The read alignments were sorted by chromosome coordinates using SAMtools (v1.9) [99]. The gene expression levels (TPM) were inferred using StringTie (v2.1.4) [100]. The read counts were called using featureCounts (v2.0.1) [101]. The differentially expressed genes were determined using R package “*limma*” [102] and “edgeR” [103].

To identify potential upstream regulators and downstream effects enriched in each T‐cell condition, the results of differential expression analysis were uploaded to the Ingenuity Pathway Analysis (IPA) software (Qiagen). To assess the functional states of each T‐cell condition, we employed the R package TCellSI (T Cell State Identifier) (v1.2.0), which generated T Cell State Scores for each condition.

### 4.13 Animals and In Vivo Procedures

All animal experiments were performed according to protocols approved by The University of Texas MD Anderson Cancer Center Institutional Animal Care and Use Committee. Housing, husbandry, and care of the mice met or exceeded the minimum requirements of the Animal Welfare Act and the Guide for the Care and Use of Laboratory Animals (eighth edition). Disease development and progression were closely monitored, and mice with metastases were euthanized as soon as we noticed signs of discomfort in the mice or when the largest dimension of a tumor reached 5 mm, according to our approved protocol. In none of the experiments were these limits exceeded.

### 4.14 Tyrosine Hydroxylase Knockout Orthotopic Tumor Model and Stereotactic Trigeminal Ganglion Injection

*Th* KO mice were generated as previously described (Jackson 2012). Briefly, a loxP site was inserted into the BsaB1 site located 600 bp 5 from the transcription start site of the *Th* gene. Another loxP site and a Frt‐flanked selectable SvNeo gene were inserted into the Kpn1 site in the first intron of the *Th* gene. The targeting construct contained 12 kb of the 5 flanking sequence and 7 kb of the 3 flanking sequences as well as a Pgk‐DTA gene for negative selection. The linearized DNA was electroporated into AB1 embryonic stem cells. Correctly targeted events (20% of the total) were detected by Southern blot of EcoR1‐digested DNA using a probe at 3 of the short arm of the targeting construct. One clone transmitted the targeted allele in the germline. Mice bearing the targeted allele were bred with FLPer mice expressing the FLIP recombinase to remove the Frt‐SvNeo cassette. After removal of the FLPer gene, the mice were backcrossed to C57BL/6J mice for several generations, resulting in male and female experimental animals. For routine genotyping of the conditional allele, two PCR primers amplifying the 150 bp region for the WT Th allele and 200 bp region for the *Th^flox/flox^* allele were used. To distinguish WT (*Th^+/+^*), hemizygous (*Th^flox/+^*), and homozygous mice (*Th^flox/flox^*), PCR genotyping of DNA extracted from mouse tails was performed using primers a and c, yielding a single 1000 bp band for *Th^floxf/lox^* mice, a single 900 bp band for T*h^+/+^* mice, and two bands for *Th^flox/+^* mice. Primer sequences were as follows: a, 5‐GTTGCAGGCTGTGTCTTC‐3; b, 5‐CCAGTGTATGTGCTGGCAC‐3; and c, 5‐GGACCCACAGAAGCCTGGCA‐3. PCR was first incubated at 98°C for 2 min, followed by 32 cycles of 98°C for 30 s, 56°C for 30 s, and 72°C for 1 min.

One week prior to implantation of murine HNSCC cells (MOC2), stereotactic AAV‐CAG ‐iCre or empty AAV vector injections were performed in the right side of the trigeminal ganglia. One week after trigeminal ganglia injection, 1 × 10^4^ murine HNSCC cells (MOC2, harboring nonsense *TP53* mutation) [41] suspended in 20 µL serum‐free medium containing Matrigel were injected into the right buccal mucosa and observed three times per week. Mice were divided into two groups: a *Th* WT (n = 10) and a *Th* KO group (n = 10). Tumor growth was assessed at the indicated time points, and tumor volume was calculated using the following formula: V = L × W^2^ × 0.5, where V is the tumor volume (mm^3^), L is the tumor length (mm), and W is the tumor width (mm).

### 4.15 Single‐Cell Preparation for Mass Cytometry

Single‐cell suspensions were prepared from freshly collected tumor samples using enzymatic digestion and a gentleMACS Dissociator (Miltenyi Biotec, #130‐096‐730), according to the manufacturer's instructions. Briefly, tumors were mechanically homogenized using a Dounce tissue homogenizer and enzymatically digested using a combination of enzyme A (12.5 µL), enzyme D (100 µL), and enzyme R (10 µL) in serum‐free RPMI 1640 medium. Tumors in the digestion buffer were then incubated at 37°C for 40 min, RPMI 1640 was used to quench the enzymatic reaction, and the tumors were filtered (70 µm). After centrifugation, cell debris was removed by using the Debris Removal Solution (Miltenyi Biotec, #130‐109‐398) and resuspended with PBS. Tumor‐infiltrating leukocytes were then sorted using mouse CD45‐microbeads according to the manufacturer's instructions (Miltenyi Biotec, #13‐052‐301).

### 4.16 Mass Cytometry

Cells were incubated with antibodies against CD16/CD32 for 10 min at room temperature to block Fc receptors. Cells were then stained with metal‐conjugated antibodies against surface markers (Fluidigm) on ice for 30 min, followed by viability staining using cisplatin (5 µM) for 1 min at room temperature. Samples were then washed with PBS containing 10% FBS, followed by fixation and permeabilization with Fixation/Permeabilization Buffer (Thermo Fisher). Cells were then incubated with metal‐conjugated antibodies against intracellular proteins (Fluidigm), washed, and then stained with Ir^191/193^ DNA intercalator (Fluidigm). Samples were stored at 4°C until acquisition. Immediately before acquisition, samples were washed and resuspended in water containing EQ Four Element Calibration Beads (Fluidigm). Samples were acquired on a Helios mass cytometer (Fluidigm). Data were first pre‐processed using the R package “premessa” to perform beads‐based normalization. Normalized data were analyzed on FlowJo (BD Biosciences), using manual gating for event length, viability, lineage markers, and phenotypic markers. Gated populations were then visualized using t‐SNE or bivariate plots.

### 4.17 Multiplex Immunofluorescence taining

Multiplex immunofluorescence staining was conducted with the Opal System (Akoya Biosciences) on the fully automated Xmatrx Elite system (BioGenex), following the manufacturer's instructions. Slides underwent automated baking, dewaxing, endogenous peroxidase blocking, antigen retrieval, and protein blocking. This was followed by a series of cycles of primary antibody and HRP‐condugated secondary polymer incubation, and Opal fluorophore deposition. Between cycles, antibody stripping was performed using heat‐induced epitope retrieval. In the last cycle, signal amplification was achieved with a DIG‐based detection step after HRP‐polymer incubation, followed by heat exposure and fluorophore deposition. Nuclei were counterstained with DAPI. Imaging was performed with a multispectral imaging system. The following panels of primary antibodies and their corresponding Opal fluorophores were used in this study: Panel 1: Anti‐beta 2 adrenergic receptor ‐ ADBR2 antibody [EPR707(N)] (Abcam, ab182136) (1:100)/Opal 520 (1:100); CXCL10 Recombinant rabbit monoclonal antibody (10H11L3) (Invitrogen, 701225) (1:50) /Opal 570 (1:100); and Pan‐keratin (Type I) (E6S1S) rabbit monoclonal antibody (Cell Signaling, 83957) (1:1500)/Opal 690 (1:100). Panel 2: Anti‐neurofilament heavy polypeptide antibody ‐ NFH (Abcam, ab8135) (1:4000)/Opal 520 (1:200); Anti‐tyrosine hydroxylase antibody ‐ TH (Millipore Sigma, AB152) (1:500)/Opal 620 (1:100); CXCL10 recombinant rabbit monoclonal antibody (10H11L3) (Invitrogen, 701225) (1:20)/Opal 780 (1:50); Anti‐beta 2 adrenergic receptor ‐ ADRB2 antibody [EPR707(N)] (Abcam, ab182136) (1:100)/Opal 570 (1:100).

### 4.18 In Silico Analysis of ROC1 Tumors

RNA‐seq data from ROC1 tumors are accessible in the Gene Expression Omnibus (GEO) under accession GSE201722. To infer transcription factor activity from this RNA‐seq data, we employed decoupleR version 2.16.0, using the CollecTRI database (https://github.com/saezlab/CollecTRI) as the source of transcription factor–target gene interactions.

### 4.19 Statistical Analysis

To perform the analyses, we utilized the JMP Pro 18 software. We used the One‐way ANOVA test followed by the Tukey–Kramer HSD post‐hoc test or the T‐test for pairwise comparisons to compare categorical and continuous variables. To determine correlations between continuous variables, we used the Pearson correlation test. To compare the frequency of T‐cell cytotoxicity under different interventions in co‐culture conditions, we used a mixed‐effects model (REML) to account for repeated measures, T‐cell: tumor cell ratios, and intervention types. Fixed effects included T‐cell: tumor cell ratios, intervention types, and their interaction, while individual cell cultures were treated as random factors. Significance was determined if the p‐value from the Fixed Effects Test for the interaction between the “intervention group” and “T‐cell: tumor cell ratio” was below 0.05. Post‐hoc Tukey's test was then used for pairwise comparisons between groups at specific T‐cell: tumor cell ratios. Tumor growth curves were also compared using a mixed‐effects model (REML), followed by post hoc comparisons for each time point using Sidak's multiple comparison test.

## Funding

T.X. is supported by the MD Anderson Cancer Center Institutional Research Grant (600382 30 124924 2). J.N.M. is the Alando J. Ballantyne Distinguished Chair of Head and Neck Surgery, The University of Texas Anderson Cancer Center, Houston, TX, USA. Work in the Talbot laboratory is supported by the Canadian Institutes of Health Research (CIHR; Grants 407016, 461274, 461275), the Canada Foundation for Innovation (44135), a Canadian Cancer Society Emerging Scholar Research Grant (708096), the Knut and Alice Wallenberg Foundation (KAW 2021.0141, KAW 2022.0327), the Swedish Research Council (2022‐01661), the Natural Sciences and Engineering Research Council of Canada (RGPIN‐2019‐06824) and NIH/NIDCR (R01DE032712). G.A.C. is the Felix L. Haas Endowed Professor in Basic Science and the Charles B. Barker Endowed Chair at The University of Texas Anderson Cancer Center, Houston, TX, USA. Work in the Calin laboratory is supported in part by the NIDCR Grant R01DE032018, Team DOD Grant in Gastric Cancer CA200990P2 c(W81XWH‐21‐1‐0715), the G. Harold & Leila Y. Mathers Foundation, a Development Grant associated with the Brain SPORE 2P50CA127001, and by the Ben and Catherine Ivy Foundation Grant. This work was supported by National Institute of Health, National Cancer Institute Grant R37 1R37CA242006‐01A1 (M. Amit), Stiefel family Discovery award (M. Amit), Institutional Research Grant (M. Amit), R01 DE032018 (NIDCR) funding (M. Amit), and by Disruptive Science Moonshot award, MDACC (M. Amit).

## Conflicts of Interest

The authors declare no conflicts of interest.

## Supporting information




**Supporting File**: advs73623‐sup‐0001‐TableS1.xlsx.

**Supporting File**: advs73623‐sup‐0002‐TableS2.xlsx.

**Supporting File**: advs73623‐sup‐0003‐TableS3.xlsx.

**Supporting File**: advs73623‐sup‐0004‐TableS4.xlsx.

**Supporting File**: advs73623‐sup‐0005‐TableS5.xlsx.

**Supporting File**: advs73623‐sup‐0006‐TableS6.xlsx.

**Supporting File**: advs73623‐sup‐0007‐TableS7.xlsx.

**Supporting File**: advs73623‐sup‐0008‐SupplS8.pdf.

## Data Availability

The data that support the findings of this study are available from the corresponding author upon reasonable request.
