# Supplementary material for: β‐Adrenergic Signaling Promotes Anti‐Tumor Immunity in TP53‐mutant Oral Squamous Cell Carcinoma
Source: Adv Sci (Weinh). 2026 Feb 10;13(35):e16859. doi: 10.1002/advs.202516859 (PMC13292172; doi:10.1002/advs.202516859)
Supplement: Supplementary file 8 — Supporting File: advs73623‐sup‐0008‐SupplS8.pdf. [file ADVS-13-e16859-s001.pdf]

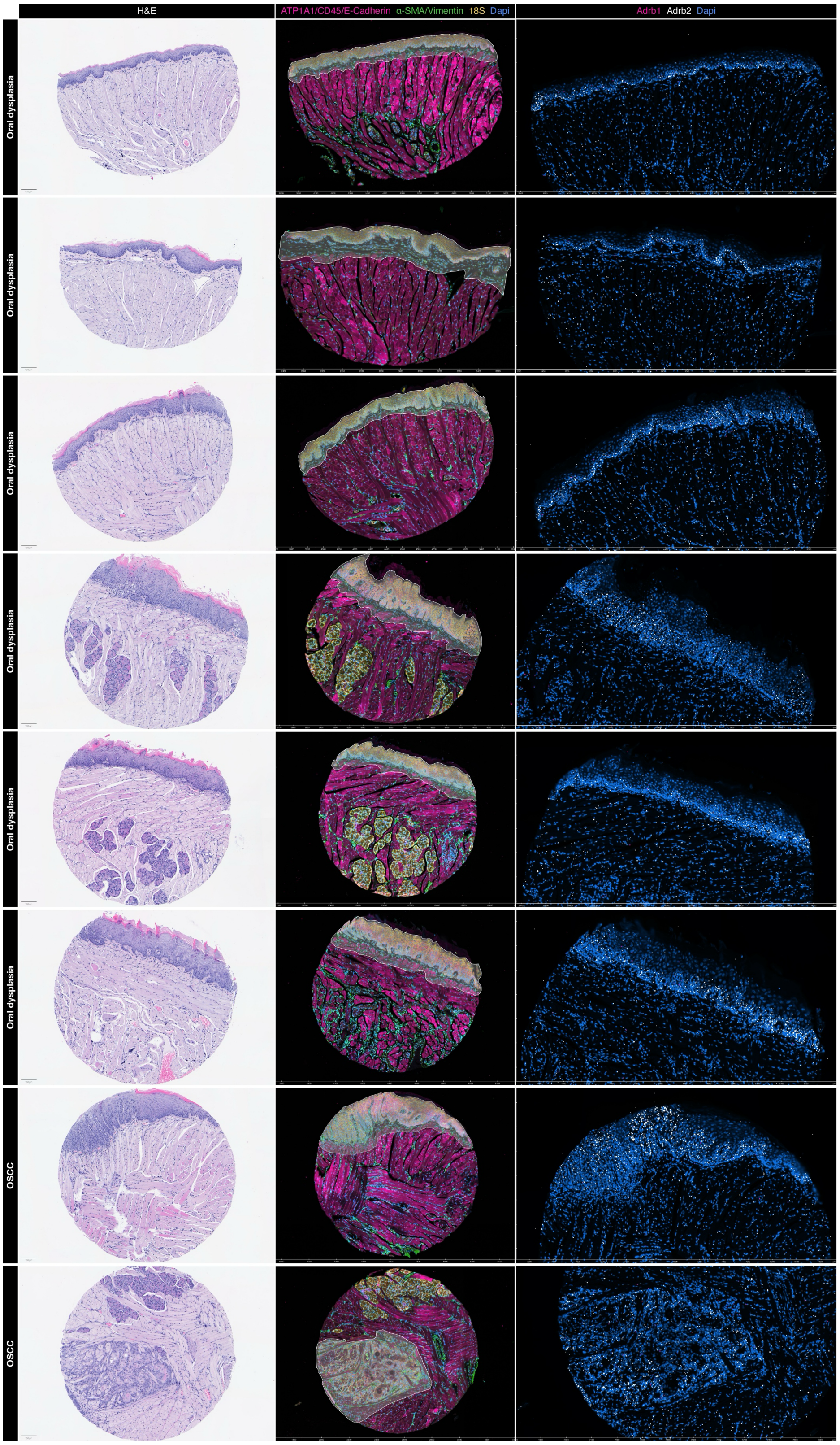

Supplementary Figure 1 – Oral mucosa lesions from mice treated with 4-NQO. We utilized eight oral lesions obtained from animals subjected to chemically-induced oral carcinogenesis using 4-NQO. Among these, two samples were identified as oral carcinoma, while the remaining samples represented oral premalignant lesions exhibiting dysplasia. These specimens were subjected to spatial transcriptome analysis utilizing the Xenium platform, with tissue labelling performed in accordance with the markers listed in the middle column. The spatial transcriptome analysis revealed widespread expression of *Adrb2* in oral epithelial cells across all subjects, whereas *Adrb1* expression was observed infrequently.

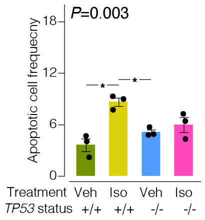

Supplementary Figure 2 – Apoptotic cell frequency in PCI13 cell lines monoculture. *TP53*$^{+/+}$ and *TP53*$^{-/-}$ PCI13 cells were cultured in monoculture (without co-cultured T cells) in the presence of isoprenaline or vehicle control, for 3 h, before flow cytometry assessment of caspase-3 activity. Data show T cell-mediated cytotoxicity, represented as a percentage of PCI-13 cells expressing cleaved caspase-3. Error bars represent the standard error of the mean. One-way ANOVA followed by the Tukey-Kramer HSD test for pairwise comparison; *P<0.05.

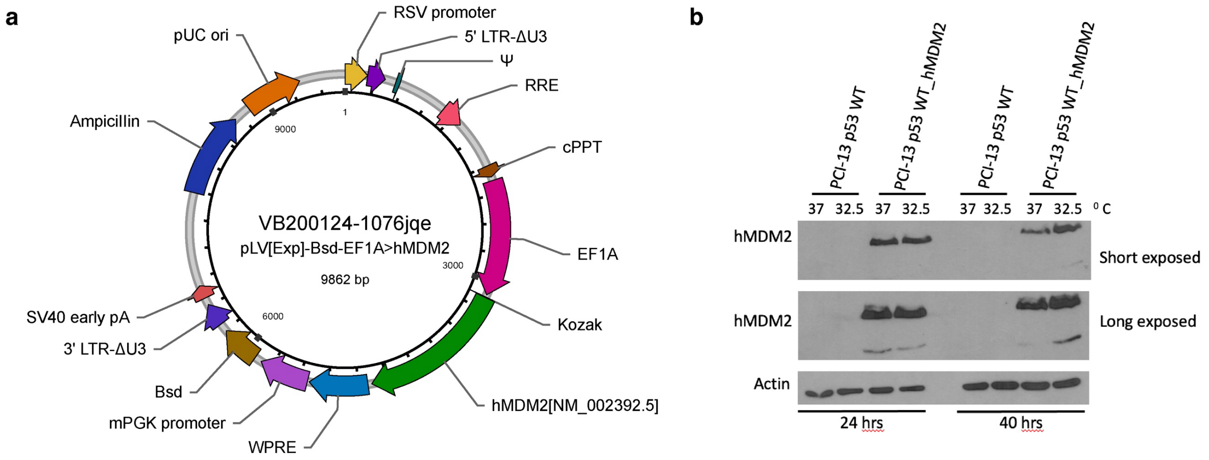

Supplementary Figure 3 – MDM2 expression in *TP53*$^{+/+}$ PCI13 cell lines. a) Viral vector employed for exogenous expression of MDM2 in *TP53*$^{+/+}$ PCI13 cell lines. **b)** Western blot analysis demonstrates MDM2 protein expression post-vector administration.

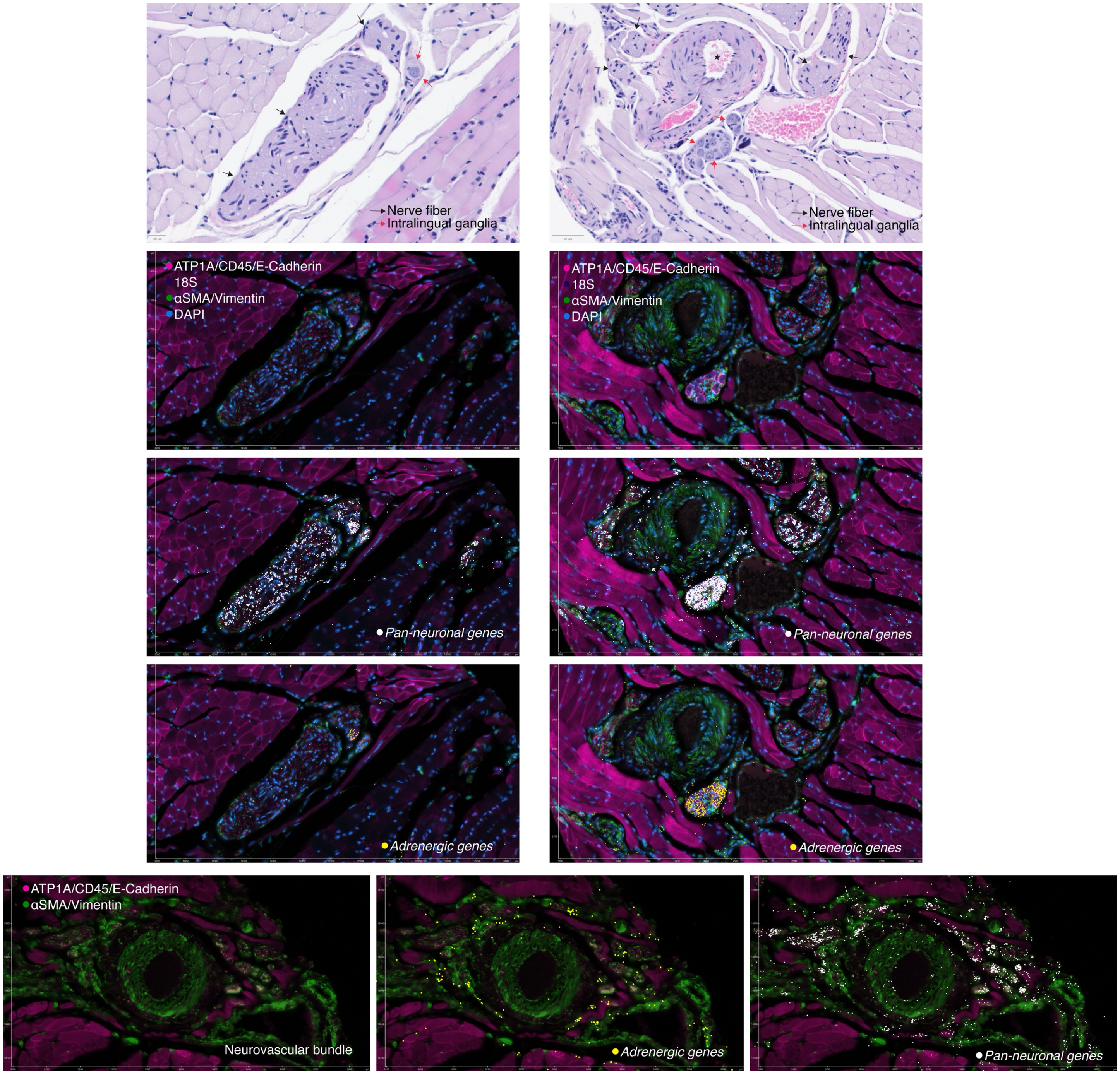

Supplementary Figure 4 – Development of gene expression signature for identification of peripheral nerves using Spatial transcriptome data. To develop a gene expression signature for detecting peripheral nerves in oral mucosa tissue from mice, we focused our analysis of spatial transcriptome data on the oral submucosa layers, specifically in areas with large nerve fibers (black arrows) and neurovascular bundles. The pan-neuronal signature (*Calca, Cdh19, Gap43, Gfra1, Gpr37l1, Lgi4, Mag, Plp1, Pmp22, Uchl1*), shown as white dots, was abundant in all large nerve fibers and in the small fibers of neurovascular bundles. The adrenergic signature (*Dbh, Slc18a2, Slc6a2, Th*), shown as yellow dots, was plentiful in intralingual ganglia (red arrows) and around neurovascular bundles.

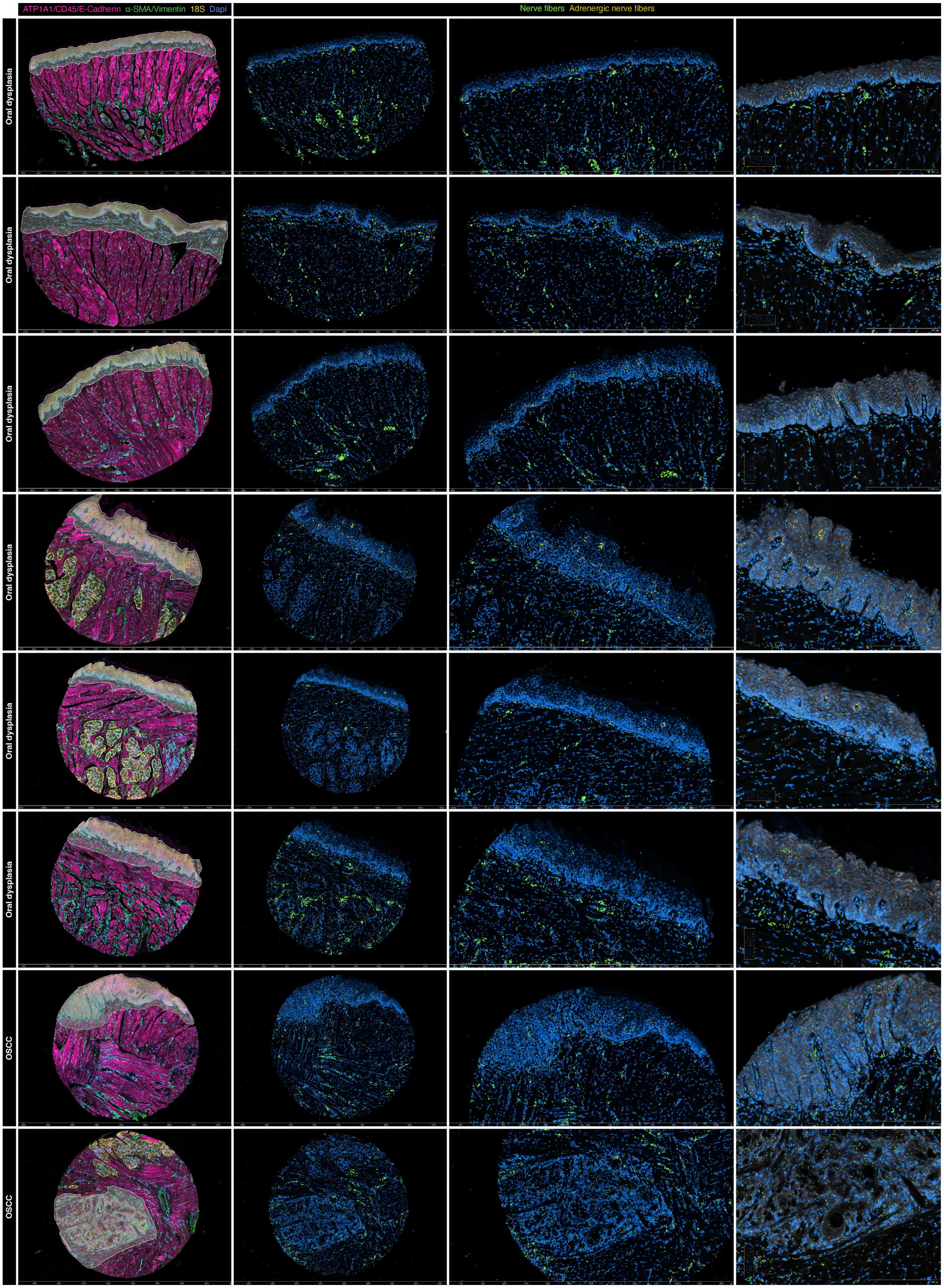

Supplementary Figure 5 – Spatial transcriptome assessment of peripheral nerves in the oral mucosa of 4-NQO-treated animals. The initial column images depict the regions designated for the evaluation of gene expression in each lesion. The subsequent images illustrate the expression of pan-neuronal genes (green) and adrenergic-specific genes (yellow) in each oral lesion. No differences in the distribution or density of nerve fibers are observed across the samples.

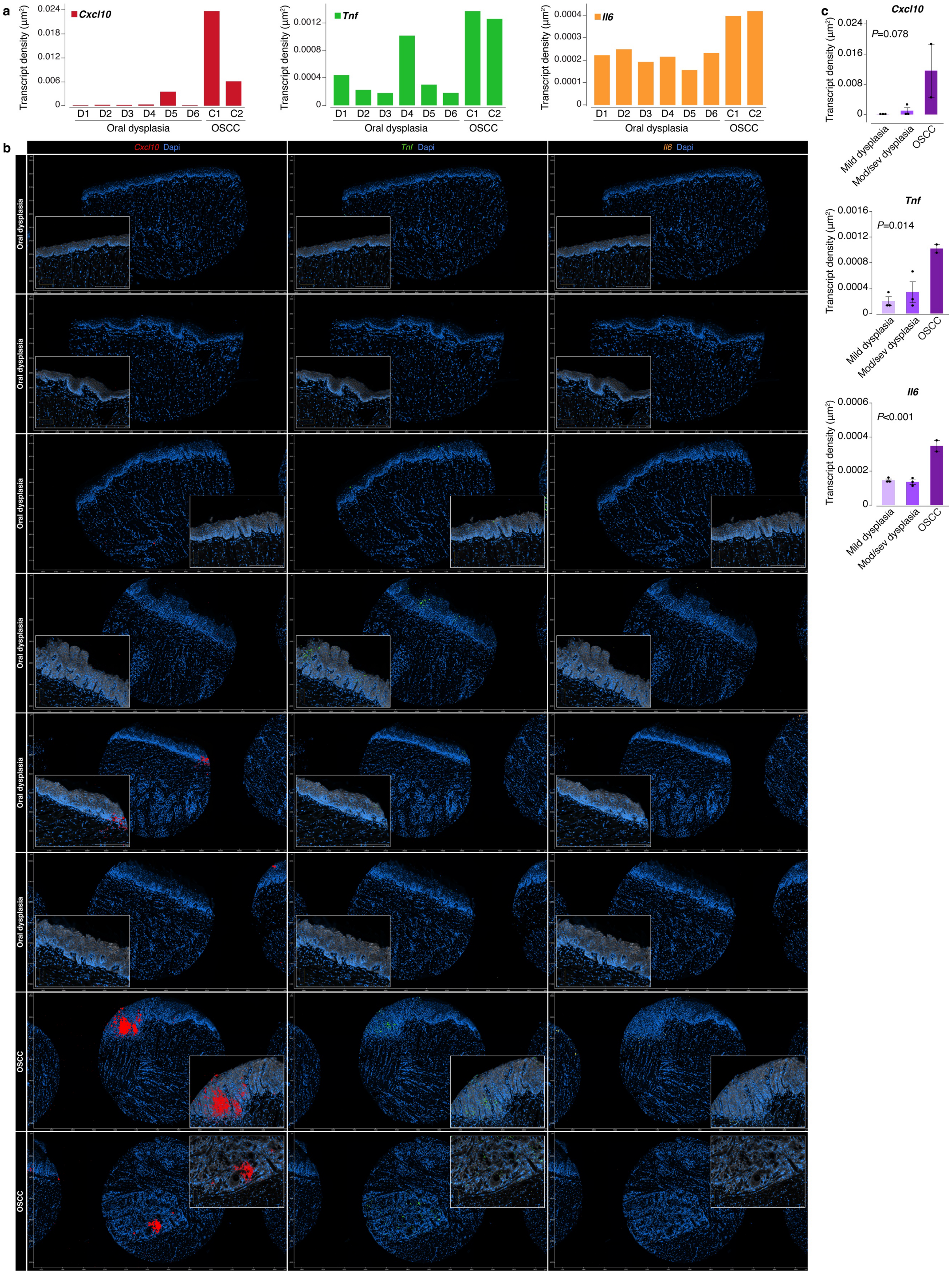

Supplementary Figure 6 – Spatial transcriptome analysis reveals cytokines that are highly expressed in cancer cells after isoprenaline treatment. a) The barplots represent the measured transcript density per μm^2 for of *Cxcl10*, *Tnf*, and *Il6* genes in the epithelial layer of all oral lesions. **b)** The images illustrate the expression levels of *Cxcl10*, *Tnf*, and *Il6* across all oral lesions evaluated in this study. **c)** Quantification of *Cxcl10*, *Tnf*, and *Il6* expression in the epithelial region of the oral mucosa lesions from 4-NQO-treated animals by spatial transcriptome analysis, according to the tissue histology. Differences were assessed using one-way ANOVA followed by the Tukey-Kramer HSD test for pairwise comparisons; $*P < 0.05$, $**P < 0.001$, $***P < 0.0001$.

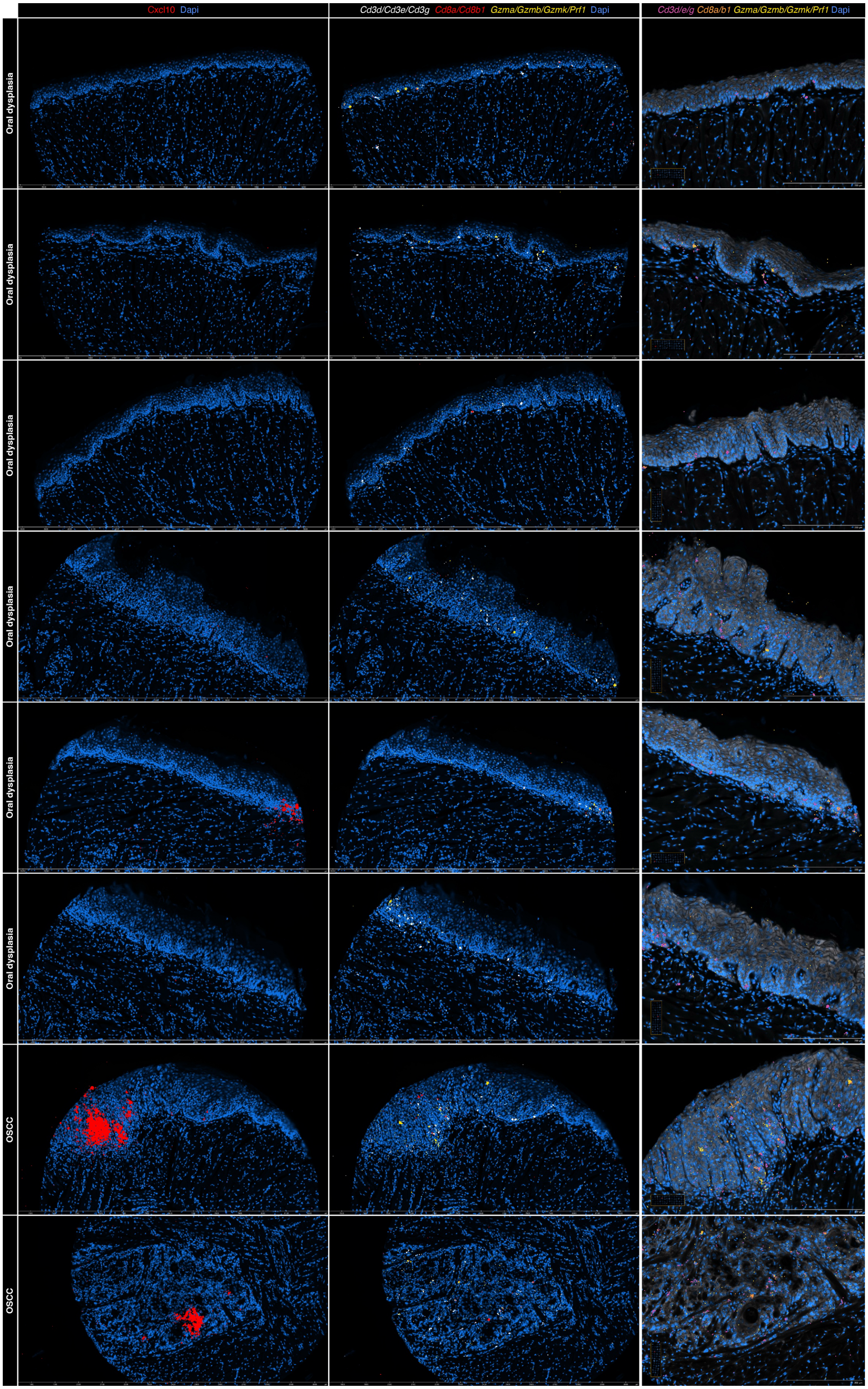

Supplementary Figure 7 – Spatial transcriptome analysis shows the correlation between Cxcl10 expression and T cell infiltration along the oral mucosa. The images depict the expression levels of *Cxcl10* and demonstrate the enrichment of T cell-associated genes in regions with elevated *Cxcl10* expression.
